# A plant plasma-membrane H^+^-ATPase promotes yeast TORC1 activation via its carboxy-terminal tail

**DOI:** 10.1038/s41598-021-83525-1

**Published:** 2021-02-26

**Authors:** Elie Saliba, Cecilia Primo, Nadia Guarini, Bruno André

**Affiliations:** Molecular Physiology of the Cell, Université Libre de Bruxelles (ULB), 6041 Biopark, Gosselies Belgium

**Keywords:** Nutrient signalling, Membrane proteins

## Abstract

The Target of Rapamycin Complex 1 (TORC1) involved in coordination of cell growth and metabolism is highly conserved among eukaryotes. Yet the signals and mechanisms controlling its activity differ among taxa, according to their biological specificities. A common feature of fungal and plant cells, distinguishing them from animal cells, is that their plasma membrane contains a highly abundant H^+^-ATPase which establishes an electrochemical H^+^ gradient driving active nutrient transport. We have previously reported that in yeast, nutrient-uptake-coupled H^+^ influx elicits transient TORC1 activation and that the plasma-membrane H^+^-ATPase Pma1 plays an important role in this activation, involving more than just establishment of the H^+^ gradient. We show here that the PMA2 H^+^-ATPase from the plant *Nicotiana plumbaginifolia* can substitute for Pma1 in yeast, to promote H^+^-elicited TORC1 activation. This H^+^-ATPase is highly similar to Pma1 but has a longer carboxy-terminal tail binding 14–3–3 proteins. We report that a C-terminally truncated PMA2, which remains fully active, fails to promote H^+^-elicited TORC1 activation. Activation is also impaired when binding of PMA2 to 14–3–3 s is hindered. Our results show that at least some plant plasma-membrane H^+^-ATPases share with yeast Pma1 the ability to promote TORC1 activation in yeast upon H^+^-coupled nutrient uptake.

## Introduction

TORC1 (Target of Rapamycin Complex 1) is a highly conserved kinase complex playing a pivotal role in controlling cell growth in probably all eukaryotic organisms^1–3^. When active, TORC1 stimulates cell growth by phosphorylating a wide variety of effector proteins that promote anabolic functions (e.g. RNA and protein synthesis, ribosome biogenesis). Other proteins phosphorylated by TORC1 inhibit autophagy or stress resistance mechanisms, and such functions are thus stimulated when TORC1 activity is low. TORC1-mediated coordination of anabolic, catabolic, and stress resistance functions is now recognized as crucial for growth control and cell survival. Furthermore, many reports suggest an association between TORC1 dysfunction and diseases including cancers^2,4^.

TORC1 control has been best studied in human cells. Upstream signals controlling mTORC1 (mechanistic or mammalian TORC1) in these cells include amino acids, nucleotides, growth factors, the energy status of the cell, and stress conditions^1,2^. A protocol widely used to study mTORC1 activation consists in starving cells of amino acids to cause mTORC1 inhibition and then resupplying the lacking amino acids to re-stimulate mTORC1. Studies using this approach have shown that activation of mTORC1 by amino acids requires a complex of two GTPases, namely RagA or B and RagC or D, which recruits mTORC1 to the lysosome. There, mTORC1 is stimulated by another small GTPase, RheB, which responds to diverse signals including growth factors^5–7^. Subsequent studies have unraveled the central role played in this pathway by cytosolic amino acid sensors (Sestrin, Castor, Samtor) controlling the Rag heterodimer via GATOR regulatory complexes^5^. Uptake of amino acids similarly activates TORC1 in yeast cells growing on a poor nitrogen source such as proline^6,7^. Initial TORC1 activation under such conditions requires Gtr1 and Gtr2, homologous to RagA/B and C/D, respectively^8^. Yet the amino acid sensors found in human cells are not conserved in yeast^5^. To date, the only TORC1-controlling amino acid sensing system to have been characterized is the leucine-tRNA synthetase, which acts through regulation of Gtr1^9^.

**Figure 1:**
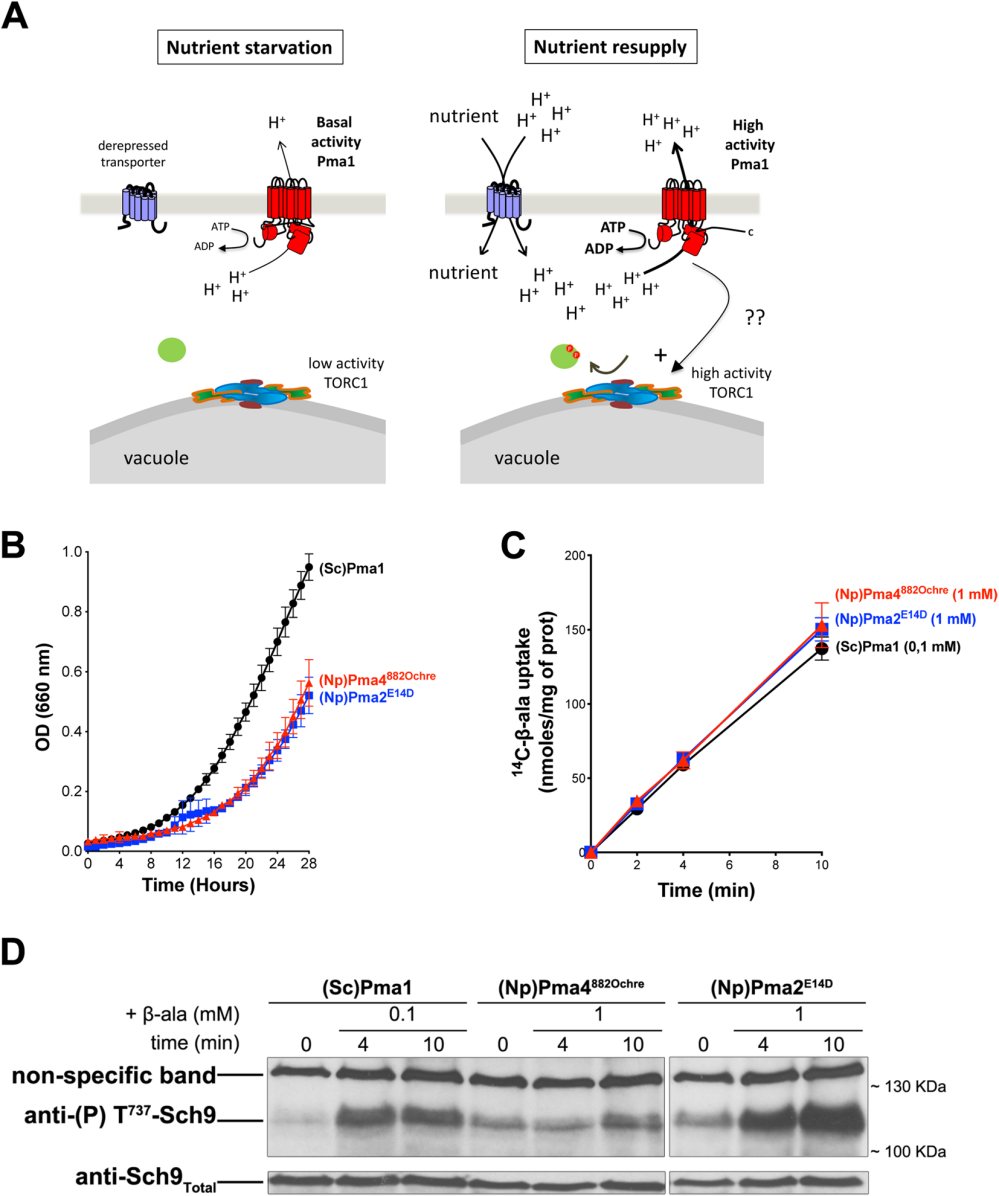
The plant H^+^-ATPase PMA2 promotes TORC1 activation in yeast. (**A**) Model of TORC1 activation upon H^+^ influx. Left. In cells starved of a specific nutrient, TORC1 activity is low and typically, a high-affinity H^+^ symporter of the limiting nutrient is strongly derepressed (e.g. Gap1, Pho84, or Sul1 in cells starved of nitrogen, phosphate, or sulfur, respectively). In these cells, the plasma-membrane Pma1 H^+^-ATPase displays basal activity. Right. When cells are resupplied with the limiting nutrient, a strong nutrient-uptake-coupled influx of H^+^ occurs. This rapidly stimulates the activity of Pma1, which extrudes excess H^+^. In this high-activity state, Pma1 also plays an active role in transient re-activation of TORC1, possibly via signaling. (**B**) *GAL1p-PMA1 pma2Δ* cells expressing, from two plasmids, either (Sc)Pma1, (Np)PMA4^882Ochre^, or (Np)PMA2^E14D^ along with HA-NPR1, were grown on Gluc NH_4_^+^ medium in a microplate reader for 28 h. Data points represent averages of the OD at 660 nm of two biological replicates; error bars represent SD. (**C**) Strains as in B were grown on Gluc NH_4_^+^ medium. After a shift to Gluc proline medium for four hours, [^14^C]-β-alanine (0.1 or 1 mM) was added to the medium before measuring the incorporated radioactivity at various times. Average values of three biological replicates are shown, and error bars correspond to SD. (**D**) Strains and growth conditions as in C. Cells were collected before and 4 and 10 min after addition of β-alanine (0.1 or 1 mM). Crude extracts were prepared and immunoblotted with anti-(P) T^737^-Sch9 and anti-Sch9_Total_ antibodies. The detected signals are from the same gel and exposure times were identical. Two parts of the gel were grouped for presentation convenience. The original blot is presented in Fig. S3.

In a recent study we found that the signal eliciting Gtr1/2-dependent TORC1 activation upon amino-acid uptake into yeast cells is the H^+^ influx coupled to the transport reaction catalyzed by amino acid/H^+^ symporters^7^. The H^+^-coupled uptake of other nutrients such as cytosine or fructose likewise triggers rapid TORC1 stimulation, and the same effect is elicited by treating cells with the protonophore carbonyl cyanide-4-(trifluoromethoxy)phenylhydrazone (FCCP). As cells starved of a specific nutrient typically derepress high-affinity H^+^-coupled transporters of this nutrient, we proposed that the H^+^ influx constitutes a general signal for initial TORC1 reactivation upon relief from diverse starvation conditions^7^. This stimulation of TORC1 is transient and followed by a more sustained activation still observed in a *gtr1Δ* mutant and proposed to be promoted by an increase of internal glutamine^6^. Yeast TORC1 is also stimulated via Gtr1/2 upon inactivation of the vacuolar V-ATPase, which causes an increase of H^+^ in the cytosol^7^. As the plasma-membrane H^+^-ATPase Pma1 is typically activated under acidic intracellular conditions^10^, we investigated its role in TORC1 activation in response to H^+^-coupled amino acid uptake or H^+^ increase in the cytosol. We replaced Pma1 with a functional plant plasma-membrane H^+^-ATPase, namely PMA4 of *Nicotiana plumbaginifolia*^11^, C-terminally truncated as this form displays higher activity than the full-length protein. In this system, despite an uptake of amino acid or cytosolic H^+^ increase equivalent to that observed in Pma1-expressing cells, TORC1 was not stimulated^7^. In contrast, we did observe proper sustained activation of TORC1 upon incubation of cells with NH_4_^+^, a process not dependent on Gtr1/2 and likely induced by NH_4_^+^ assimilation into amino acids^6,7^. The yeast plasma-membrane H^+^-ATPase Pma1 thus appears to play an essential and specific role in H^+^-influx-elicited TORC1 stimulation (Fig. 1A). This role involves more than just establishment of the H^+^ gradient, possibly signaling. Furthermore, independent studies have revealed intracellular pH as an important signal for control of cell growth in yeast^12,13^.

Multiple plasma-membrane H^+^-ATPase isoforms exist in plants and have been best characterized in *Arabidopsis thaliana* and *N. plumbaginifolia*^14–16^. As in fungi, these H^+^-ATPases establish an H^+^ gradient that energizes the plasma membrane. Although very similar to yeast Pma1, plant H^+^-ATPases differ from it by a longer carboxy-terminal tail (C-tail). This cytosolic region is an autoinhibitory domain whose action appears to be assisted by the extreme N-terminus of the protein^14–16^. The negative effect exerted by this C-tail on the activity of plant H^+^-ATPases is modulated by phosphorylation of several residues in this region. For instance, phosphorylation of the penultimate Thr residue, occurring in both yeast and plants via unknown kinases, promotes binding of 14–3–3 protein dimers to the last ~ 30 residues of the H^+^-ATPase, resulting in neutralization of self-inhibition^17–19^. Association of the C-tail with 14–3–3 proteins also promotes organization of the H^+^-ATPase into wheel-like hexamers, considered to be the fully active form of the enzyme^20,21^. Phosphorylation of other C-tail residues either impedes binding of 14–3–3 s (likely via steric hindrance), thereby reducing the H^+^-ATPase activity, or exerts an opposite, stimulatory effect^14,16^. Phosphorylation of C-tail residues is controlled by multiple physiological signals, including external pH, blue light, and phytohormones. It is now assumed that the activity of plant H^+^- ATPases is regulated by practically all factors known to regulate plant growth^14^. Yet whether these enzymes are involved in controlling TORC1 in plant cells remains unexplored.

In this paper, we report that the *N. plumbaginifolia* PMA2 H^+^-ATPase can substitute for Pma1 in promoting TORC1 activation in response to an H^+^ influx or increase. Furthermore, this stimulation of TORC1 depends on the C-tail of the H^+^-ATPase and is modulated by its association with 14–3-3 proteins. Our results suggest that at least some plant plasma-membrane H^+^-ATPases share with yeast Pma1 the ability to promote TORC1 activation upon H^+^ influx or increase.

## Results

### The plant H^+^-ATPase PMA2 promotes TORC1 activation in yeast

To analyze TORC1 activation in yeast expressing a plant plasma-membrane H^+^-ATPase, we typically use a *GAL1p-PMA1 pma2Δ* strain, i.e. a strain where the *PMA1* gene is placed under the control of the galactose-inducible, glucose-repressible *GAL1* promoter and the *PMA2* gene, encoding a second, poorly expressed H^+^-ATPase, is deleted. The strain contains a plasmid expressing a plant H^+^-ATPase gene under the control of the *PMA1* promoter. Transformed cells are initially grown on a minimal buffered (pH 6.1) glucose medium (Gluc) containing NH_4_^+^ as sole nitrogen source. These conditions allow optimal growth and high TORC1 activity. The cells are then shifted for 4 h to the same medium except that NH_4_^+^ is replaced with proline. As proline is a poor nitrogen source, TORC1 activity is reduced. The cells are then treated to cause an influx of H^+^ in order to restimulate TORC1. This can be triggered, for instance, by adding β-alanine (β-Ala). This amino acid is efficiently transported by the general amino-acid permease (Gap1), an amino acid/H^+^ symporter. Furthermore, it cannot be used as a nitrogen source, and its uptake does not lead to an increase of any other amino acid^7^. Using this protocol, we previously compared the effects of β-Ala uptake in cells expressing the endogenous Pma1 and in cells where Pma1 was replaced with PMA4^882ochre^, a truncated version of the *N. plumbaginifolia* H^+^-ATPase PMA4, lacking the last 71 residues. At equivalent β-Ala uptake, TORC1 was found to be activated in Pma1-expressing cells but not in PMA4^882ochre^-expressing cells^7^.

This result prompted us to examine whether other plant H^+^-ATPases are likewise unable to promote TORC1 activation in response to an H^+^ influx. We tested *N. plumbaginifolia* PMA2, which belongs to another H^+^-ATPase subfamily and differs from PMA4 in that it remains active at neutral pH^11^. Furthermore, PMA2 is stimulated under acidic conditions in tobacco (*N. tabacum*) BY-2 cells^22^. Yeast cells expressing PMA2 as the sole functional plasma-membrane H^+^-ATPase grow relatively slowly at pH 6.5, because of limited activity^23^. We thus expressed a PMA2 mutant with the E14D substitution in the N-terminal tail. As this substitution makes the H^+^ pump less sensitive to self-inhibition by its C-tail, PMA2^E14D^ is hyperactive and supports faster growth^24^. The PMA2^E14D^ variant used in our experiments also contains a His tag added between the N-terminal residues 3 and 4 and shown not to interfere with H^+^-ATPase activity^25^. On the buffered Gluc NH_4_^+^ medium, yeast cells expressing PMA4^882ochre^ or PMA2^E14D^ grew at a similar rate (Fig. 1B), though 1.6-fold slower compared to Pma1 cells. This illustrates, in keeping with previous observations^11,24^, that neither plant H^+^-ATPase is as active as the endogenous yeast H^+^-ATPase. After a shift of the cells to proline for 4 h, radiolabeled β-Ala (1 mM) was added to the cultures. Cells expressing PMA4^882ochre^ or PMA2^E14D^ were found to take it up at a similar rate (Fig. 1C), but as this rate was lower than that of Pma1 cells, we reduced the external concentration of β-Ala added to the Pma1 cells in order to reach equivalent uptakes in all three strains (Fig. 1C). We next collected culture samples, prepared cell extracts, and detected phosphorylation of Sch9 kinase residue Thr737, a classical readout of TORC1 activity^26^. In the Pma1 cells, uptake of β-Ala caused a net increase of Sch9 phosphorylation (Fig. 1D), and this increase was not observed if the cells were pre- or post-treated with rapamycin (RAP) (Fig. S1), as expected. In PMA4^882ochre^ cells, basal Sch9 phosphorylation was a bit higher but, as previously shown, it increased only very slightly upon equivalent β-Ala uptake (Fig. 1D)^7^. In PMA2^E14D^ cells, basal Sch9 phosphorylation was also higher, but remarkably, phosphorylation markedly increased upon β-Ala transport (Fig. 1D) unless the cells were treated with RAP (Fig. S1). This increase in phosphorylation was even reproducibly higher than in Pma1 cells. Hence, in contrast to PMA4^882ochre^, PMA2^E14D^ can promote TORC1 activation in yeast in response to an H^+^-influx-coupled amino acid uptake.

### The carboxy-terminal tail of plant PMA2 is required for TORC1 activation in yeast

**Figure 2:**
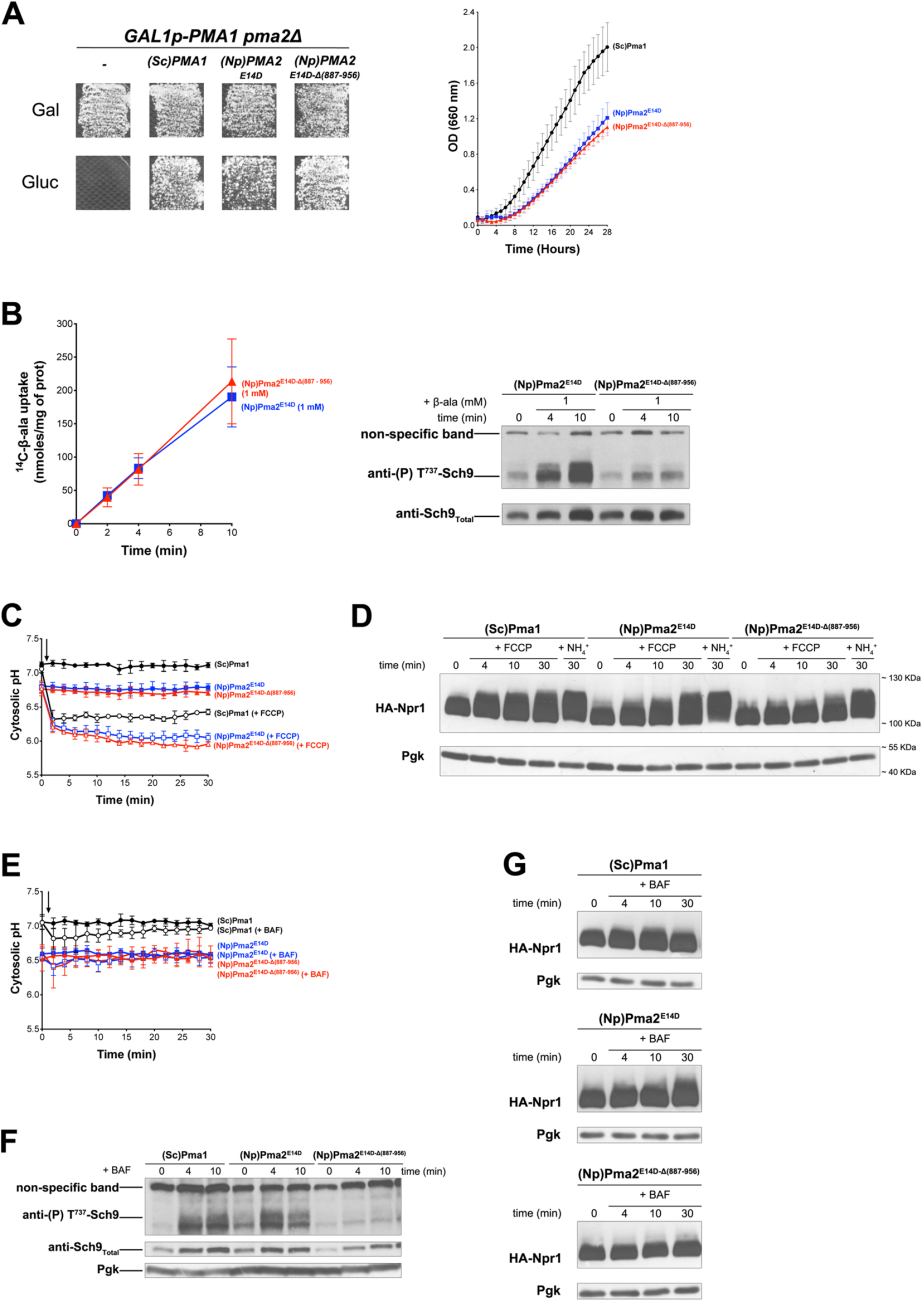
The carboxy-terminal tail of plant PMA2 is required for TORC1 activation in yeast. (**A**) Left. *GAL1p-PMA1 pma2Δ* cells transformed with a plasmid expressing (Sc)PMA1*,* (Np)PMA2^E14D^, (Np)PMA2^E14D-Δ(887-956)^, or no H^+^-ATPase (−) were grown for three days on solid medium with NH_4_^+^ as nitrogen source and Gal or Gluc as carbon source. Right. *GAL1p-PMA1* *pma2Δ* cells expressing from plasmids either (Sc)Pma1*,* (Np)PMA2^E14D^*,* or (Np)PMA2^E14D-Δ(887-956)^ along with HA-NPR1 were grown on Gluc NH_4_^+^ medium in a microplate reader for 28 h. Data points represent averages of the OD at 660 nm of three biological replicates; error bars represent SD. (**B**) Left. *GAL1p-PMA1 pma2Δ* cells expressing from plasmids (Np)PMA2^E14D^ or (Np)PMA2^E14D-Δ(887-956)^ along with HA-NPR1, were grown on Gluc NH_4_^+^ medium. After a shift to Gluc proline medium for four hours, [^14^C]-β-alanine (1 mM) was added to the medium before measuring the incorporated radioactivity at various times. Average values of three biological replicates are shown, and error bars correspond to SD. Right. Strains and growth conditions as in the left panel. Cells were collected before and 4 and 10 min after addition of β-alanine (1 mM). Crude extracts were prepared and immunoblotted with anti-(P) T^737^-Sch9 and anti-Sch9_Total_ antibodies. (**C**) *GAL1p-PMA1 pma2Δ* cells expressing, from plasmids, either (Sc)Pma1*,* (Np)PMA2^E14D^*,* or (Np)PMA2^E14D-Δ(887-956)^ along with pHluorin were grown on Gluc NH_4_^+^ medium. After a shift to Gluc proline medium for 4 h, the cytosolic pH was measured at various times during growth with (open symbols) or without (filled symbols) addition of FCCP (20 µM) starting at 1 min (indicated by an arrow on the graph). Average values of three biological replicates are shown, and error bars correspond to SD. (**D**) *GAL1p-PMA1 pma2Δ* cells expressing, from plasmids, either (Sc)Pma1*,* (Np)PMA2^E14D^*,* or (Np)PMA2^E14D-Δ(887-956)^ along with HA-Npr1 were grown on Gluc NH_4_^+^ medium. After a shift to Gluc proline medium for four hours, cells were collected before and 4, 10, and 30 min after addition of FCCP (20 µM) or 30 min after addition of NH_4_^+^ (5 mM). Crude extracts were prepared and immunoblotted with anti-HA and anti-Pgk antibodies. (**E**) Strains and growth conditions as in D, except that cells were treated (open symbols) or not (filled symbols) with bafilomycin A (BAF) (1 µM). (**F**) Cell extracts analyzed in G (only those collected at 4 and 10 min) were migrated in a separate gel and immunoblotted with anti-(P) T^737^-Sch9, anti-Sch9_Total_ and anti-Pgk antibodies. (**G**) Same strains and growth conditions as in D, except that cells were treated with bafilomycin A (BAF) (1 µM). The detected signals are from the same gel and exposure times were identical. Strains are presented in separate panels for convenience. Original blots of figure panels B, D, F and G are presented in Fig. S3.

The amino acid sequences of *N. plumbaginifolia* PMA2 and PMA4 are highly similar over their entire lengths, including their C-tails (81% identity). A major difference between PMA4^882ochre^ and PMA2^E14D^, used in the above experiment, is that PMA2^E14D^ has a full-length C-tail, whereas PMA4^882ochre^ lacks the last 71 residues of this region. We thus reasoned that the C-tail of plant H^+^-ATPases might play an important role in TORC1 activation. To assess this hypothesis, we isolated a truncated Pma2^E14D^ variant lacking the equivalent last 70 residues (887-956). Previous studies have shown that similar C-tail truncations in PMA2 (from residue 871, 881, or 891) make the H^+^-ATPase more active in yeast, because self-inhibition is lost^24,27^. Furthermore, the initial PMA2^E14D^ variant is already much less sensitive to C-tail-mediated autoinhibition. Like PMA2^E14D^, the PMA2^E14D-Δ887–956^ mutant proved able to substitute for Pma1 as regards growth on solid Gluc NH_4_^+^ medium (pH 6.1) (Fig. 2A), and cells expressing PMA2^E14D^ or PMA2^E14D-Δ887-956^ grew at similar rates in the equivalent liquid medium (Fig. 2A). We then tested these cells for the ability to activate TORC1 upon β-Ala uptake. After growth on NH_4_^+^ and transfer to proline, PMA2^E14D^ and PMA2^E14D-Δ887-956^ cells took up the radiolabeled amino acid at equivalent rates (Fig. 2B), suggesting that the full-length and truncated PMA2^E14D^ variants established similar H^+^ gradients at the plasma membrane. Remarkably, analysis of Sch9 phosphorylation revealed that TORC1 activation in response to β-Ala uptake, observed in PMA2^E14D^ control cells, was strongly reduced in cells expressing the truncated form PMA2^E14D-Δ887-956^ (Fig. 2B). This suggests that the C-tail of *N. plumbaginifolia* PMA2 plays an important role in activation of yeast TORC1 elicited by H^+^-influx-coupled amino acid uptake.

We next sought to confirm this finding under other conditions known to cause an H^+^ influx or an H^+^ increase in the cytosol. One such condition is addition of the protonophore FCCP^7^. It has been found, however, that Sch9 phosphorylation does not increase upon FCCP addition, likely because TORC1 activity toward Sch9 is inhibited under various stress conditions and because acidification of the cytosol is stressful for the cell^12,28^. FCCP-elicited TORC1 stimulation can nevertheless be detected by analyzing Npr1, another kinase whose phosphorylation markedly increases upon TORC1 stimulation^29^, in a manner that seems less sensitive to stress^28^. TORC1 activation in response to either β-Ala uptake or FCCP addition depends on the Gtr1/2 GTPase heterodimer, but after prolonged incubation with FCCP, it is sometimes possible to detect a limited Gtr1/2-independent activation of TORC1. This suggests that an additional pathway, whose triggering signal remains unknown, can activate TORC1 in FCCP-treated cells^7^. Before FCCP addition, cells expressing PMA2^E14D^ or PMA2^E14D-Δ887-956^ showed a similar cytosolic pH, slightly lower than the neutral pH observed in Pma1 cells (Fig. 2C). This further illustrates that neither PMA2 form fully compensates for the lack of endogenous Pma1 as regards control of the intracellular pH. Upon FCCP addition, as expected, the cytosolic pH rapidly dropped to values close to that of the buffered medium. Then, in PMA2^E14D^ and PMA2^E14D-Δ887-956^ cells, it tended to show a further gradual decline, whereas in Pma1 cells it was more stable (Fig. 2C). We next analyzed the influence of FCCP on HA-Npr1 phosphorylation (Fig. 2D and Fig. S2). In FCCP-treated Pma1 cells, HA-Npr1 showed decreased electrophoretic mobility indicative of TORC1-promoted phosphorylation^29^, unless the cells were treated with RAP. Cells expressing PMA2^E14D^ displayed a similar RAP-sensitive response to FCCP, confirming that this H^+^-ATPase can promote yeast TORC1 activation in response to H^+^ influx. In contrast, PMA2^E14D-Δ887-956^ cells, despite similar cytosol acidification, showed only limited increase in HA-Npr1 phosphorylation, more pronounced 30 min after FCCP addition. These results support the above conclusion that PMA2^E14D^ promotes TORC1 activation in a C-tail-dependent manner. As a further control, we analyzed HA-Npr1 after addition of NH_4_^+^ (Fig. 2D). Assimilation of NH_4_^+^ into cells growing on proline results in sustained TORC1 activation which, unlike the transient TORC1 stimulation elicited by an H^+^ influx, proceeds via a Gtr1/2-independent mechanism^6,7^. In both PMA2^E14D^ and PMA2^E14D-Δ887-956^ cells, addition of NH_4_^+^ was found to induce HA-Npr1 phosphorylation, indicating that TORC1 can be stimulated via a different pathway in PMA2^E14D-Δ887-956^ cells. In other words, the absence of H^+^-influx-elicited TORC1 activation in cells expressing PMA2^E14D-Δ887-956^ does not seem due to some general defect in TORC1 stimulation. This conclusion is further supported by the observation that these cells do not grow more slowly than cells expressing the full-length PMA2^E14D^.

We next analyzed TORC1 activation upon inhibition of the vacuolar V-ATPase by bafilomycin A (BAF). We have previously reported that this treatment, causing an increase in cytosolic H^+^ and Pma1 stimulation^10^, also promotes TORC1 activation and that this response does not occur if Pma1 is replaced with plant PMA4^882ochre^ ^7^. Upon BAF addition to Pma1 cells, acidification of the cytosol was barely detectable (Fig. 2E), likely because activated Pma1 can efficiently compensate an H^+^ increase in the cytosol. As previously reported^7^, this BAF treatment caused a net increase of Sch9 phosphorylation (Fig. 2F). The more acidic cytosolic pH of PMA2^E14D^ and PMA2^E14D-Δ887-956^ cells did not detectably change upon BAF addition (Fig. 2E), suggesting that both PMA2 forms might also be capable of compensating an H^+^ increase in the cytosol. In PMA2^E14D^ cells, BAF caused increased phosphorylation of both Sch9 and HA-Npr1 (Fig. 2F,G, and Fig. S2C), again illustrating the ability of the full-length enzyme to support TORC1 activation in response to an H^+^ increase. In cells expressing PMA2^E14D-Δ887-956^, however, TORC1 was not activated upon BAF addition (Fig. 2F,G).

In conclusion, removal of the last 70 residues of the C-tail of PMA2^E14D^ strongly impairs the ability of this plant H^+^-ATPase to promote yeast TORC1 activation under diverse conditions of H^+^ increase. As this C-tail extension is highly conserved in PMA4, this plant H^+^-ATPase isoform belonging to another subfamily might also be able to promote TORC1 activation in yeast. We were unable to test this hypothesis, however, because full-length PMA4 does not efficiently substitute for Pma1 in promoting yeast cell growth^11^.

### The inability of C-terminally truncated PMA2 forms to activate TORC1 is not associated with reduced H^+^ pumping activity

**Figure 3:**
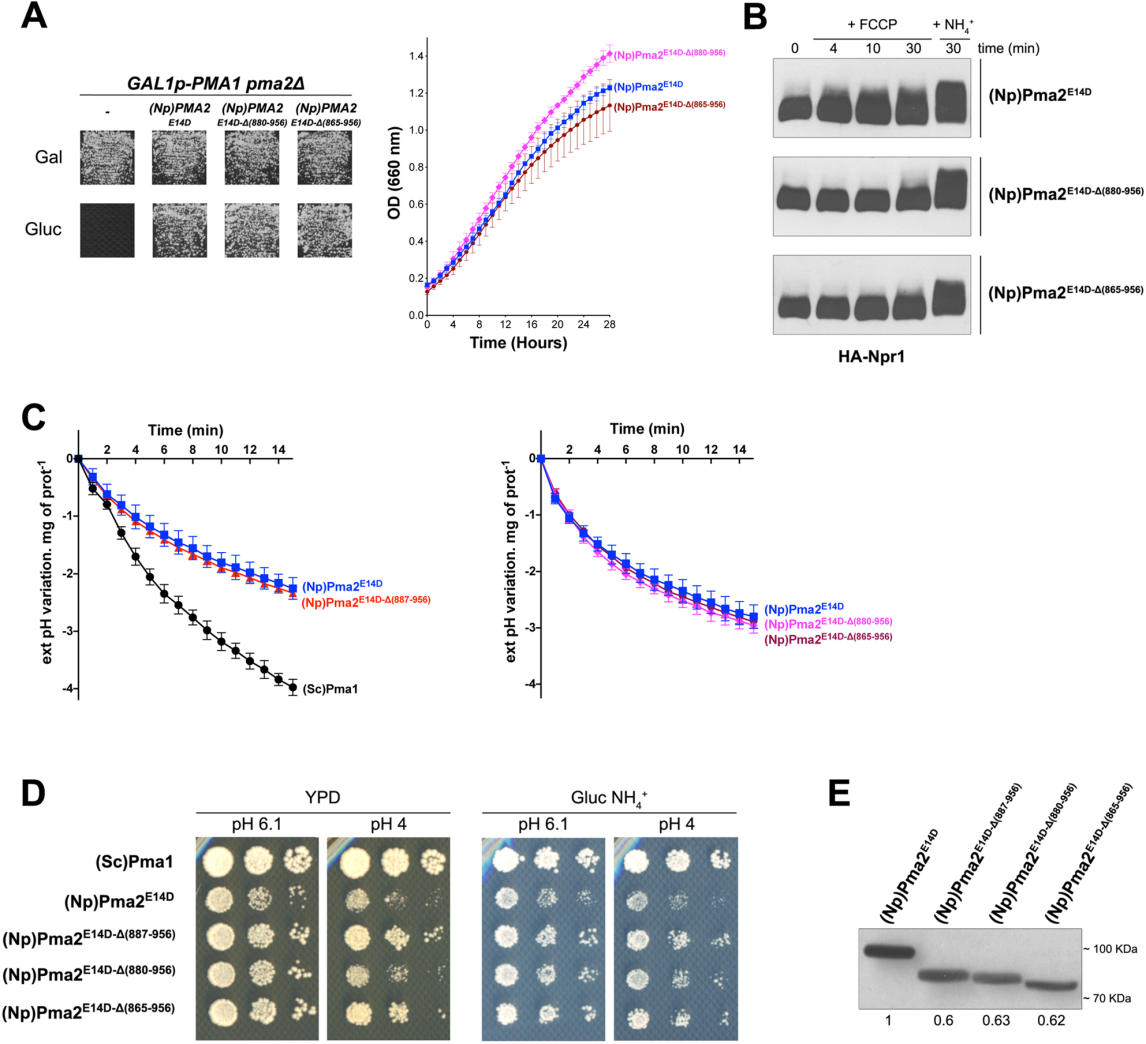
The inability of C-terminally truncated PMA2 forms to activate TORC1 is not associated with reduced H^+^ pumping activity. (**A**) Left. *GAL1p-PMA1 pma2Δ* cells expressing, from a plasmid, either (Np)PMA2^E14D^*,* (Np)PMA2^E14D-Δ(880-956)^, (Np)PMA2^E14D-Δ(865-956)^, or no H^+^-ATPase (-) were grown for 3 days on solid medium with NH_4_^+^ as sole nitrogen source and Gal or Gluc as carbon source. Right. *GAL1p-PMA1 pma2Δ* cells expressing, from plasmids, either (Np)PMA2^E14D^, (Np)PMA2^E14D-Δ(880-956)^, or (Np)PMA2^E14D-Δ(865-956)^ along with HA-NPR1 were grown on Gluc NH_4_^+^ medium in a microplate reader for 28 h. Data points represent averages of the OD at 660 nm of two biological replicates; error bars represent SD. (**B**) The same cells as in panel A (right) were grown on Gluc NH_4_^+^ medium. After a shift to Gluc proline medium for four hours, culture samples were collected before and 4, 10, and 30 min after addition of FCCP (20 µM) or 30 min after addition of NH_4_^+^ (5 mM). Crude extracts were prepared and immunoblotted with the anti-HA antibody. The signals are from the same gel and exposure times were identical. Strains are presented in separate panels for convenience. (**C**) Left. *GAL1p-PMA1 pma2Δ* cells expressing, from plasmids, either (Sc)Pma1*,* (Np)PMA2^E14D^*,* or (Np)PMA2^E14D-Δ(887-956)^ along with HA-NPR1 were grown on Gluc NH_4_^+^ medium. Acidification by these cells of the external medium was measured as described in Materials and Methods. Right. Same as in the left panel, except that cells expressing (Np)PMA2^E14D^, (Np)PMA2^E14D-Δ(880-956)^, or (Np)PMA2^E14D-Δ(865-956)^ were analyzed. Average values of three biological replicates are shown, and error bars correspond to SD. (**D**) *GAL1p-PMA1 pma2Δ* cells expressing, from plasmids, either (Sc)Pma1, (Np)PMA2^E14D^, (Np)PMA2^E14D-Δ(887-956)^, (Np)PMA2^E14D-Δ(880-956)^, or (Np)PMA2^E14D-Δ(865-956)^ along with HA-NPR1 were spotted in two-fold serial dilutions on solid rich (YPD) or Gluc NH_4_^+^ (pH 6.1 or 4) medium and incubated for four days. (**E**) *GAL1p-PMA1 pma2Δ* cells expressing, from plasmids, either (Np)PMA2^E14D^, (Np)PMA2^E14D-Δ(887-956)^, (Np)PMA2^E14D-Δ(880-956)^ or (Np)PMA2^E14D-Δ(865-956)^ along with HA-NPR1 were grown on Gluc NH_4_^+^ medium. After a shift to Gluc proline medium for four hours, the cells were collected. Crude extracts were prepared and immunoblotted with the anti-polyHis antibody. Direct Blue 71 staining (Sigma-Aldrich) was used for quantitative comparisons and signals were normalized to the signal of (Np)PMA2^E14D^-expressing cells. Original blots of figure panels B and E are presented in Fig. S3.

It has been reported that the C-tail of plant H^+^-ATPases includes several sequences that self-inhibit the H^+^ pumping activity of these enzymes^24,27,30^. We therefore reasoned that the remaining C-tail in truncated PMA2^E14D-Δ887-956^ (about 30 amino acids) might have a self-inhibitory effect on the ability of PMA2 to activate TORC1, this effect being neutralized by sequences further downstream when the C-tail is complete. For instance, a previous study describing the influence of deletions of increasing length in the C-tail of *Nicotiana plumbaginifolia* PMA2 revealed that the region between residues 869 and 872 has a limited self-inhibitory effect, which is abolished if the C-tail is truncated up to residue 869 or a position further upstream^27^. We therefore isolated two additional PMA2^E14D^ mutants with more extended C-tail deletions, from residue 880 or 865. These variants also proved able to compensate for the lack of Pma1 in promoting cell growth on both solid and liquid media (Fig. 3A), but like PMA2^E14D-Δ887–956^, both failed to promote TORC1 activation in response to an FCCP-elicited H^+^ influx (Fig. 3B). In contrast, TORC1 was efficiently activated in these strains after NH_4_^+^ addition (Fig. 3B).

The absence of TORC1 activation in cells expressing the PMA2^E14D^ variant with the shortest C-tail deletion (residues 887-956) does not seem due to lower activity of the H^+^-ATPase, as β-Ala uptake into these cells and their growth rate equaled those measured in cells expressing full-length PMA2^E14D^ (Fig. 2B). Nevertheless, to further assess this possibility, we grew cells on Gluc NH_4_^+^ medium, shifted them to proline for 4 h, and collected and washed them before measuring acidification of the extracellular medium induced by Gluc addition (Fig. 3C). As expected, cells expressing the plant H^+^-ATPase acidified the medium at a lower rate (~ 1.75-fold) than cells expressing Pma1, but no significant difference was detected between cells expressing full-length PMA^2E14D^ and cells expressing the truncated PMA2^E14D-Δ887-956^ version. The same result was obtained with the two other C-terminally truncated PMA2^E14D^ variants described above (Fig. 3C).

It is well established that high plasma-membrane H^+^-ATPase activity is required for optimal growth of yeast on acidic media^10,31^. We thus also compared growth of the above-described strains on solid media adjusted to different pH values (Fig. 3D). On both rich and minimal media adjusted to pH 6.1, growth of cells expressing the full-length PMA2^E14D^ was slightly reduced as compared to cells expressing Pma1, in agreement with previous results^24^. This difference in growth, however, was less obvious in the case of cells expressing a PMA2^E14D^ variant with a C-tail truncation up to residue 887 or 865. On a more acidic medium (pH 4), the growth differences were more pronounced. Specifically, PMA2^E14D^ cells showed a more substantial growth reduction than Pma1 cells, but this effect was less accentuated for cells expressing PMA2^E14D-Δ865-956^ or PMA2^E14D-Δ887-956^. This suggests that under acidic conditions, when a highly active plasma-membrane H^+^-ATPase is needed to extrude excess H^+^, full-length PMA2^E14D^ cannot sustain optimal growth. In contrast, at least two of the three isolated truncated forms of PMA2^E14D^ support better growth under these acidic conditions. This suggests that the hyperactive PMA2^E14D^ mutant remains partially sensitive to C-tail-mediated autoinhibition, in keeping with previous conclusions^22,24^. In any case, the only effect of C-tail truncation in PMA2^E14D^ unraveled by these growth tests is an increase in activity. Lastly, cell extracts were immunoblotted to compare levels of PMA2^E14D^ and of its three truncated derivatives (Fig. 3E). Although the three variants were less abundant, the above data show that they are at least as active as PMA2^E14D^. Two of them even proved more active in cells placed on an acidic medium. Taken together, these results show that in cells expressing PMA2^E14D^ with a truncated C-tail, impaired TORC1 activation is not associated with any apparent reduction of H^+^-ATPase activity.

### Yeast TORC1 activation via the plant H^+^-ATPase PMA2 requires association of its carboxy-terminal tail with 14–3–3 proteins

Phosphorylation of Thr955, the penultimate residue of PMA2, has been shown to increase in plant cells under acidic conditions^32^. Subsequent binding of 14–3–3 proteins to a region covering the last ~ 30 amino acids of the enzyme alleviates the autoinhibition mediated by its C-tail. The T955A substitution, which abolishes binding of 14–3–3 s, thus inhibits PMA2 activity^25^. Although this inhibition is largely relieved when the protein also contains the E14D substitution, it is still observable, especially under acidic conditions^25^, as illustrated above (Fig. 3D). Furthermore, phosphorylation of the further-upstream residues Thr931 and Ser938, detected in plant cells, causes inhibition of PMA2 activity, most likely by impeding 14–3–3 protein binding^22^. This inhibitory effect is observed in yeast when these residues of PMA2 are replaced with the phosphomimetic Asp residue (T931D, S938D), but not if PMA2 contains the S938A substitution. The T931A substitution, unlike S938A, also interferes with 14–3–3 binding and PMA2 stimulation. The negative effect of these substitutions, like that of T955A, is less pronounced when they are introduced into the hyperactive PMA2^E14D^ mutant^22^. We thus examined whether these substitutions in PMA2^E14D^ might influence its ability to promote yeast TORC1 activation upon H^+^ influx.

**Figure 4:**
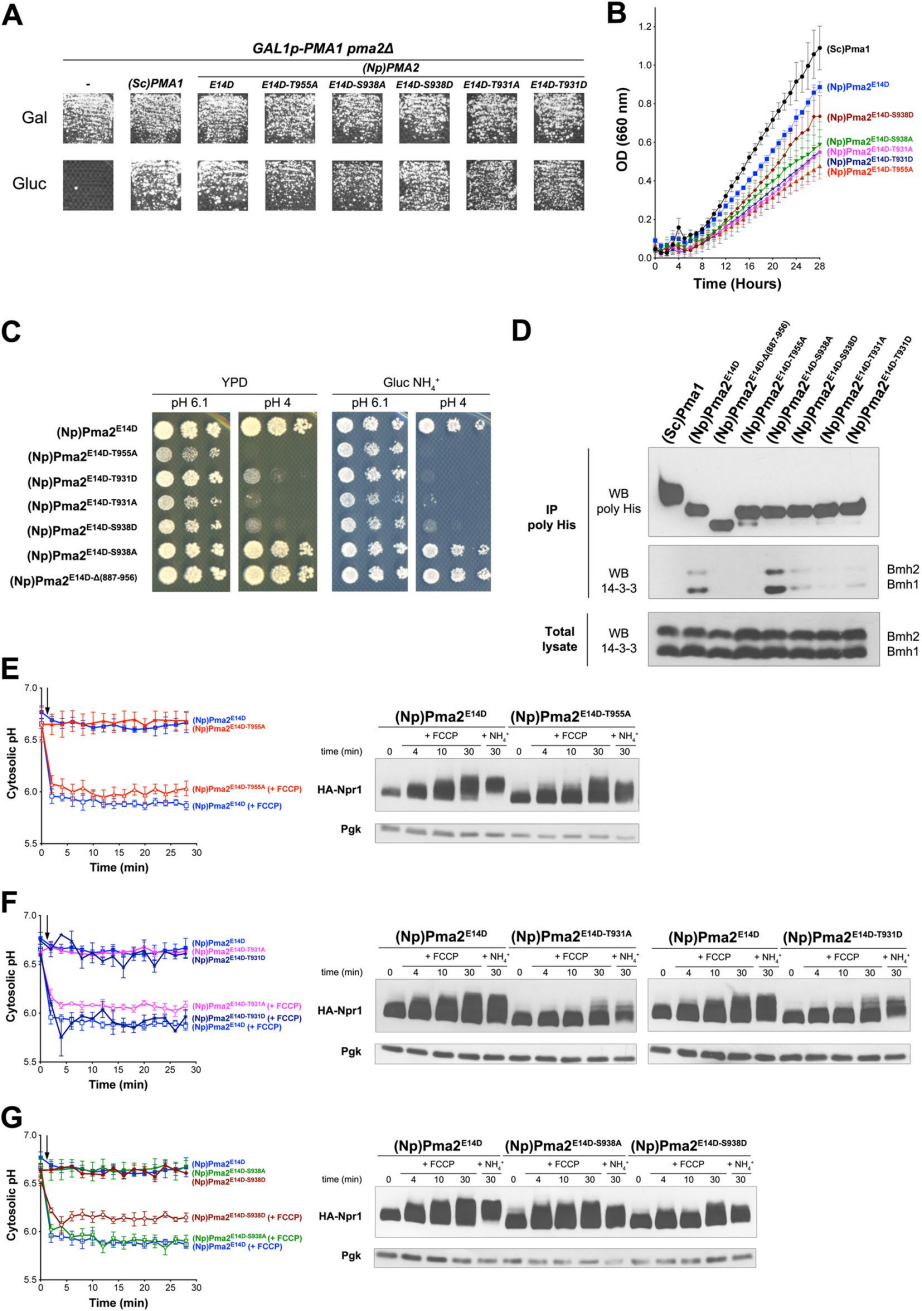
Yeast TORC1 activation via the plant H^+^-ATPase PMA2 requires association of its carboxy-terminal tail with 14-3-3 proteins. (**A**) *GAL1p-PMA1 pma2Δ* cells transformed with plasmids expressing (Sc)PMA1*,* (Np)PMA2^E14D^*,* (Np)PMA2^E14D-T955A^, (Np)PMA2^E14D-S938A^, (Np)PMA2^E14D-S938D^, (Np)PMA2^E14D-T931A^, (Np)PMA2^E14D-T931D^, or no H^+^-ATPase (-) were grown for three days on solid medium with NH_4_^+^ as nitrogen source and Gal or Gluc as carbon source. (**B**) The same cells as in A, additionally expressing HA-NPR1 from a plasmid, were grown on Gluc NH_4_^+^ medium in a microplate reader for 28 h. Data points represent averages of the OD at 660 nm of two biological replicates; error bars represent SD. (**C**) *GAL1p-PMA1 pma2Δ* cells expressing, from a plasmid, either (Np)PMA2^E14D^*,* (Np)PMA2^E14D-T955A^, (Np)PMA2^E14D-T931A^, (Np)PMA2^E14D-T931D^, (Np)PMA2^E14D-S938D^, (Np)PMA2^E14D-S938A^, or (Np)PMA2^E14D-Δ(887-956)^ were spotted in two-fold serial dilutions on solid rich (YPD) or Gluc NH_4_^+^ (pH 6.1 or 4) medium and incubated for three days. Equivalent results were obtained when the strains additionally expressed HA-Npr1. (**D**) *GAL1p-PMA1 pma2Δ* cells expressing, from plasmids, 6xHis-tagged (Sc)Pma1*,* (Np)PMA2^E14D^*,* (Np)PMA2^E14D-Δ(887-956)^, (Np)PMA2^E14D-T955A^, (Np)PMA2^E14D-S938A^, (Np)PMA2^E14D-S938D^, (Np)PMA2^E14D-T931A^, or (Np)PMA2^E14D-T931D^ along with HA-NPR1 were grown on Gluc NH_4_^+^. After a shift to Gluc proline medium for four hours, the cells were collected and lysed, and His-tagged proteins were pulled down as described in Materials and Methods. Lysates and pulled-down fractions were immunoblotted with anti-14-3-3 or anti-polyhistidine antibodies. (**E**) Left. *GAL1p-PMA1 pma2Δ* cells expressing, from plasmids, either (Np)PMA2^E14D^ or (Np)PMA2^E14D-T955A^ along with pHluorin were grown on Gluc NH_4_^+^ medium. After a shift to Gluc proline for four hours, the cytosolic pH was monitored with (open symbols) or without (filled symbols) addition of FCCP (20 µM), starting at 1 min (indicated by an arrow on the graph). Average values of three biological replicates are shown, and error bars correspond to SD. Right. Strains and growth conditions as in the left panel, except that the cells expressed HA-NPR1 instead of pHluorin. Culture samples were collected before and 4, 10, and 30 min after addition of FCCP (20 µM) or 30 min after addition of NH_4_^+^ (5 mM). Crude extracts were prepared and immunoblotted with anti-HA and anti-Pgk antibodies. (**F**,**G**) Experiments similar to those in E, except that other (Np)PMA2^E14D^ mutants were analyzed, as indicated. Original blots of figure panels D, E, F and G are presented in Fig. S3.

The PMA2^E14D^ H^+^-ATPases with an additional T955A, T931A, T931D, S938A, or S938D substitution are functional, since they were found to compensate for the absence of Pma1 as regards growth on solid Gluc NH_4_^+^ medium (Fig. 4A). These substitutions, however, reduced cellular growth (from 1.5- to twofold) in the corresponding liquid medium (Fig. 4B). Furthermore, growth of PMA2^E14D^ cells on a more acidic medium (pH 4) was strongly impaired by the T955A, T931D, T931A, and T938D substitutions but neither by the S938A substitution nor by truncation of the C-tail (Fig. 4C), in keeping with previous observations^22,25^.

We next exploited the His tag inserted into the N-terminus of these PMA2^E14D^ variants to pull down each of them from cell extracts and to assess their association with yeast 14–3-3 proteins. These proteins are encoded by two redundant genes, *BMH1* and *BMH2*^33^, and can be distinguished on immunoblots as they display different electrophoretic mobilities (Fig. 4D). In keeping with previous experiments performed with cells grown on a rich medium^34^, PMA2^E14D^ was found associated with both Bmh1 and Bmh2, and this interaction was abolished by the T955A substitution and by deletion of the last 70 residues. Interaction of PMA2^E14D^ with Bmh proteins was also strongly weakened by the T931D, T931A, and S938D substitutions. In contrast, the S938A substitution did not reduce binding to 14–3–3 s and rather tended to reinforce it (Fig. 4D), as previously reported^22^. We also analyzed cells expressing the endogenous Pma1 and found no detectable interaction with any Bmh protein (Fig. 4D).

We next compared TORC1 activation by FCCP in cells expressing PMA2^E14D^ or PMA2^E14D-T955A^. The initial cytosolic pH in these cells was similar and rapidly dropped upon FCCP addition, as expected (Fig. 4E). In PMA2^E14D^ control cells, FCCP elicited the expected increase in HA-Npr1 phosphorylation. This response was reduced in PMA2^E14D-T955A^ cells, especially in the early minutes after FCCP addition, although a slight TORC1 activation remained detectable. The T955A substitution, preventing binding of 14–3–3 s and favoring self-inhibition^25^, thus impedes TORC1 activation upon H^+^ influx. In cells expressing PMA2^E14D^ with a T931A or T931D substitution, acidification of the cytosol upon FCCP addition did not elicit a marked increase in HA-Npr1 phosphorylation, except after 30 min of treatment. This shows that these PMA2^E14D^ mutants largely fail to promote early TORC1 stimulation upon H^+^ influx (Fig. 4F). The same TORC1 stimulation defect was observed in cells expressing PMA2^E14D^ with the S938D substitution, but not when S938 was replaced with Ala (Fig. 4G). Lastly, in the above strains where TORC1 failed to be properly stimulated by FCCP, its activation was detectable after addition of NH_4_^+^, although it was lower than in PMA2^E14D^ cells. Taken together, these results indicate that substitutions in the C-tail of PMA2^E14D^ which hinder its association with 14–3-3 proteins ^22^ also impede activation of TORC1 in response to an H^+^ influx.

### Reduced expression of 14–3–3 s impedes PMA2-mediated TORC1 activation

**Figure 5:**
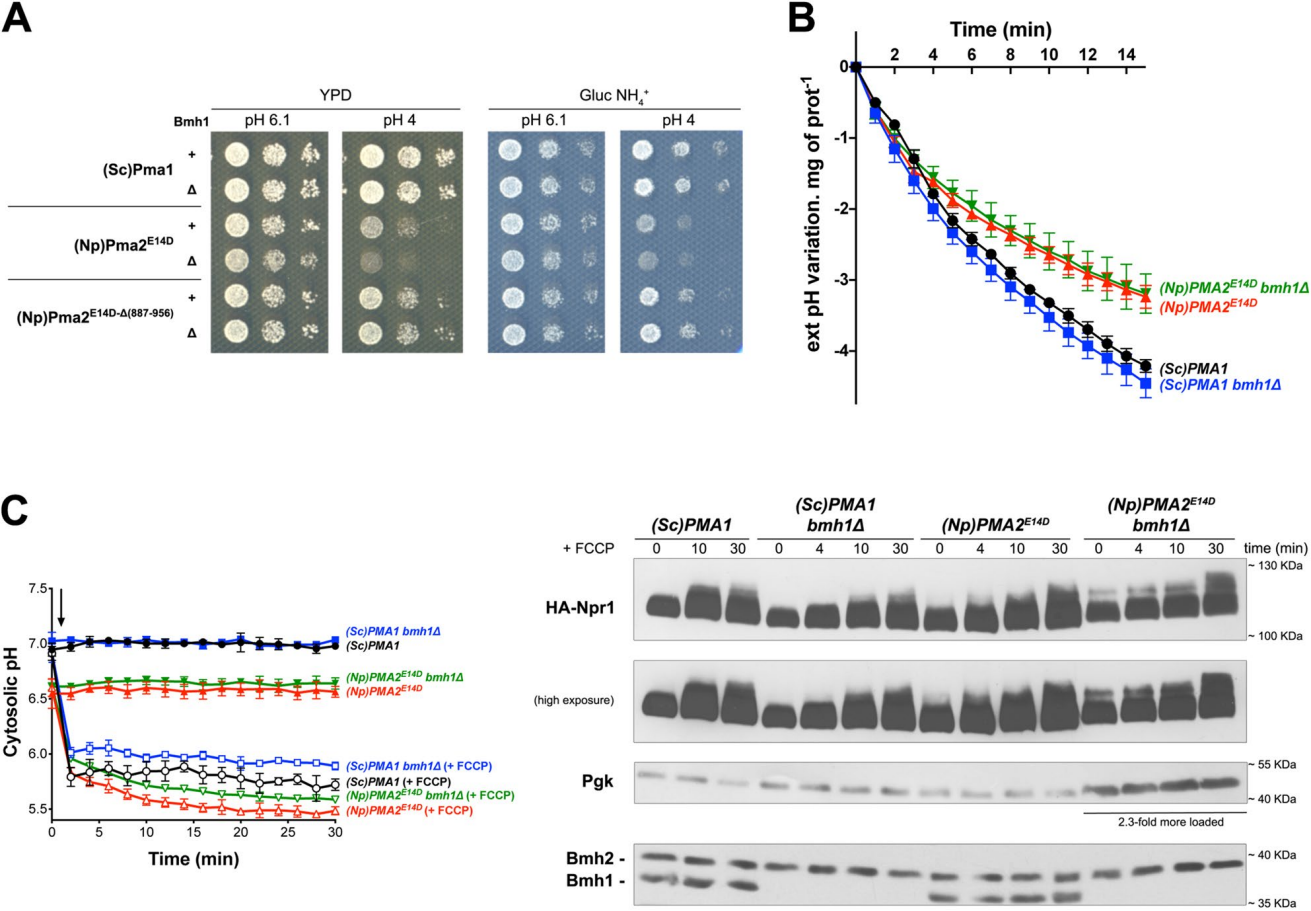
Reduced expression of 14–3–3 s impedes PMA2-mediated TORC1 activation. (**A**) *GAL1p-PMA1 pma2Δ* or *GAL1p-PMA1 pma2Δ bmh1Δ* cells expressing from a plasmid either (Sc)Pma1, (Np)PMA2^E14D^, or (Np)PMA2^E14D-Δ(887–956)^ were spotted in two-fold serial dilutions on solid rich (YPD) or Gluc NH_4_^+^ (pH 6.1 or 4) medium and incubated for three days. Similar results were obtained with the same strains additionally expressing HA-Npr1. (**B**) *GAL1p-PMA1 pma2Δ* or *GAL1p-PMA1 pma2Δ bmh1Δ* cells expressing, from plasmids, either (Sc)Pma1 or (Np)PMA2^E14D^ along with HA-NPR1 were grown on Gluc NH_4_^+^ medium. Acidification by these cells of the external medium was measured as described in Materials and Methods. Average values of three biological replicates are shown, and error bars correspond to SD. (**C**) Left. Strains and growth conditions as in B, except that the cells expressed pHluorin instead of HA-Npr1from a plasmid. After a shift to Gluc proline medium for four hours, the cytosolic pH was monitored with (open symbols) or without (filled symbols) addition of FCCP (20 µM), starting at 1 min (indicated by an arrow on the graph). Average values of three biological replicates are shown, and error bars correspond to SD. Right. Strains, growth conditions, and FCCP treatment as in B, except that the cells expressed HA-NPR1 instead of pHluorin. Cells were collected before and 4, 10, and 30 min after addition of FCCP (20 µM). Crude extracts were prepared and immunoblotted with anti-HA and anti-Pgk antibodies. For samples of cells expressing (Np)PMA2^E14D^, 2.3-fold more cell extract was loaded to compensate for the lower expression of HA-Npr1 which was systematically observed in independent transformed clones, for unclear reasons. On a separate gel, all samples were equally loaded and immunoblotted with anti-14–3-3. Original blots are presented in Fig. S3.

The above experiments confirmed that PMA2^E14D^ binds both yeast 14–3–3 proteins, Bmh1 and Bmh2^25^. These were expressed at roughly similar levels under the tested conditions (Fig. 4D). Yet PMA2^E14D^ seemed to interact more efficiently with Bmh1, as also observed in another study^22^. This prompted us to examine whether a lack of Bmh1 might influence PMA2^E14D^ activity and TORC1 stimulation upon H^+^ influx. We thus deleted the *BMH1* gene in the *GAL1p-PMA1 pma2Δ* strain and expressed in the resulting mutant the full-length and truncated versions of PMA2^E14D^ and the endogenous Pma1 as a control. We first compared the growth of these strains on rich and minimal media buffered at pH 6.1 or 4 (Fig. 5A). On all tested media, whether Bmh1 was present or not did not affect the growth of cells expressing Pma1. In PMA2^E14D^ cells, a lack of Bmh1 did not alter growth on media at pH 6.1. On the more acidic medium (pH 4) where these cells show slower growth than Pma1 cells, the absence of Bmh1 caused a further reduction of growth, most obvious on rich medium. This effect of Bmh1, however, was not observed with cells expressing the truncated form PMA2^E14D-Δ887-956^. This suggests that it is exerted through the C-tail of PMA2^E14D^. We also compared acidification of the external medium by these cells. In this experiment, however, we did not detect any influence of the *bmh1Δ* mutation, whether the cells expressed Pma1 or PMA2^E14D^ (Fig. 5B). Hence, a lack of Bmh1 seems to cause detectable effects only under acidic conditions. We finally cultivated *wild-type* and *bmh1Δ* cells expressing Pma1 or PMA2^E14D^ in Gluc NH_4_^+^ medium, transferred them to proline to reduce TORC1 activity, and then treated them with FCCP. As expected, the resulting acidification of the cytosol was similar whether Bmh1 was present or not (Fig. 5C). Analysis of HA-Npr1 phosphorylation showed that in Pma1 cells, a lack of Bmh1 did not impede TORC1 activation by FCCP. PMA2^E14D^ cells, in contrast, showed markedly reduced early activation of TORC1 when Bmh1 was not expressed (Fig. 5C). This result indicates that Bmh1 plays an important role in PMAE^E14D^-dependent TORC1 activation elicited by H^+^ influx.

## Discussion

Although TORC1 is highly conserved among eukaryotes, the physiological signals and mechanisms controlling its activity, best studied in human cells, have obviously diverged among the major eukaryotic lineages. For instance, while the three core TORC1 subunits characterized in plants (Tor kinase, Lst8, Kog1/Raptor) are highly similar to fungal and human counterparts, no plant orthologs of the RheB and Rag/Gtr small GTPases nor of their direct upstream regulators has been identified^35,36^. Furthermore, although nutrient (e.g. amino acid) availability and stress conditions also control TORC1 in plant cells, other signals more specifically related to the physiology and growth of photosynthetic organisms (sucrose, light, phytohormones) also contribute importantly to TORC1 control^3,37^.

Fungi and plant cells share a major feature distinguishing them from animal cells: their plasma membrane is energized by an H^+^ gradient established by highly conserved H^+^-ATPases^31,38^. These enzymes are very abundant, consume much ATP, and are essential to viability. At least some of them are stimulated under acidic conditions and play an important role in extrusion of excess H^+^ and control of intracellular pH^10^. Plant H^+^-ATPases are also involved in cell expansion, and this was historically explained by the acid growth theory according to which these enzymes, by acidifying the external medium, promote cell wall loosening^39^. We have recently found that diverse conditions inducing an H^+^ increase in the yeast cytosol, used in the present study and thought to stimulate the activity of the Pma1 H^+^-ATPase, elicit rapid activation of TORC1. The underlying mechanisms remain unknown, except that this activation depends on the Rag-related Gtr1/2 small GTPases^7^. That TORC1, involved in nutrient sensing, is also stimulated upon a cytosolic H^+^ increase makes sense, as a sudden H^+^ influx typically occurs when cells starved of a specific nutrient are replenished. Uptake of the limiting nutrient is indeed generally catalyzed by high-affinity H^+^ symporters that are typically derepressed during starvation (Fig. 1A).

In this study, we report that an H^+^ influx or increase also activates yeast TORC1 in cells where the Pma1 H^+^-ATPase has been replaced with the closely related *N. plumbaginifolia* PMA2 enzyme bearing the E14D substitution in its N-terminal tail (making it more active in yeast). Yet when the C-tail of PMA2^E14D^ is truncated, this TORC1 activation is impaired. This loss of TORC1 activation was not accompanied by reduced activity of the truncated PMA2^E14D^. For instance, H^+^-coupled uptake of β-alanine via the Gap1 permease was equivalent in cells expressing the complete or a truncated PMA2^E14D^ form. These cells also grew at same rates in liquid media and acidified the extracellular medium with equal efficiencies. The cytosolic pH of these cells was also equivalent, though more acidic than in *wild-type* yeast because the plant H^+^-ATPases do not fully compensate for the lack of Pma1. The only detected effect of C-tail truncation in PMA2^E14D^ was improved growth on acidic media, reflecting higher activity, in keeping with previous reports^24^. Hence, the inability of C-terminally truncated PMA2^E14D^ to activate TORC1 cannot be explained by a reduction of its H^+^-ATPase activity nor to slower growth of the cells expressing it. The simplest explanation of our observations is that the plant H^+^-ATPase plays an active role in triggering some intracellular reactions leading to TORC1 stimulation upon H^+^ influx, and does so in a C-tail-dependent manner.

Previous studies have shown that the C-tail of PMA2 and other plant H^+^-ATPases is an autoinhibitory domain. This region likely acts by physically interacting with several catalytic regions of the H^+^-ATPase^14,16,40^. It also seems assisted by the extreme N-terminus of the protein, which explains the strong resistance to self-inhibition of plant PMA2 bearing the E14D substitution^24,30^. The molecular details of this self-inhibition remain unclear, however, as the interactions of the C-tail with the rest of the enzyme have not been elucidated to date at the structural level. Self-inhibition of H^+^-ATPases by their C-tail is modulated by phosphorylation of several of Thr and Ser residues, the best characterized of which is the penultimate Thr. Phosphorylation of this Thr, by still unknown kinases, promotes binding of 14–3–3 s, which alleviates self-inhibition. Remarkably, this phosphorylation also occurs in yeast, via kinases which also remain uncharacterized^14–16^. More upstream Ser and Thr residues, when phosphorylated, either impede or stimulate binding of 14–3–3 s, thus inversely modulating the H^+^-ATPase’s activity. Phosphorylation of Ser938 in *N. plumbaginifolia* PMA2, for instance, inhibits the H^+^-ATPase by impeding binding to 14–3–3 s^22^, and studies of the AHA2 H^+^-ATPase of *A. thaliana* have shown that the equivalent phosphorylation, which has the same negative effect, involves the PKS5 kinase controlled by salt stress^40,41^. Our results show that PMA2^E14D^ mutants with substitutions hindering binding to yeast 14–3–3 s are also largely defective in H^+^-influx-triggered TORC1 activation. Furthermore, those PMA2^E14D^ derivatives which fail to interact properly with 14–3–3 s also display reduced H^+^ pumping activity, as judged by their reduced ability to sustain growth on acidic media. These observations thus indicate that when binding of 14–3–3 s to the C-tail of PMA2^E14D^ is hindered, self-inhibition by this regulatory region is more pronounced, as illustrated in previous works^14–16^, and that this correlates with a reduced ability to promote TORC1 activation upon H^+^ influx. This view is further supported by the behavior of PMA2^E14D^-expressing cells lacking Bmh1, the yeast 14–3–3 isoform to which PMA2^E14D^ more tightly binds. In these cells, TORC1 is not properly activated upon H^+^ influx. This phenotype is not observed with cells expressing the endogenous Pma1 H^+^-ATPase, which does not detectably interact with 14–3–3 s. In these Bmh1-lacking cells, furthermore, the apparent activity of PMA2^E14D^ seems only slightly reduced. This raises the interesting possibility that C-tail self-association, favored by reduced binding to 14–3–3 s, can strongly hinder TORC1 activation without markedly reducing the H^+^-ATPase’s activity, at least when PMA2 bears the E14D substitution. Yet most active PMA2^E14D^ derivatives are those lacking the C-tail (and thus unable to bind 14–3–3 s), and these proved largely unable to stimulate TORC1. The results thus suggest that the C-tail of PMA2 plays a dual role: it inhibits the H^+^-ATPase’s activity by interacting with cytosolic catalytic regions, but once this inhibition is relieved by phosphorylation of the penultimate Thr and efficient binding to 14–3–3 s, it also actively contributes to TORC1 activation. A consequence of this apparent dual role is that TORC1 activation via the H^+^-ATPase will mostly occur when the enzyme is highly active, as expected when nutrients are actively taken up by the cells and ATP is abundant enough to fuel the H^+^-ATPase.

We have not investigated the intracellular molecular events through which plasma membrane H^+^-ATPases, i.e. the endogenous Pma1 or the heterologous PMA2^E14D^, actively contribute to yeast TORC1 activation in response to an H^+^ influx or increase. Further work is clearly needed to address this question. A tentative model is that these enzymes, once stimulated under acidic conditions, for instance via a change of conformation^42^, might establish interactions with cytosolic or membrane-embedded factors. These would in turn be stimulated, for example by phosphorylation, thereby initiating a cascade of events culminating with TORC1 activation. In other words, plasma-membrane H^+^-ATPases, once stimulated by H^+^, might signal to TORC1 via a molecular pathway whose early steps, at least, would be conserved between fungi and plants. This would explain why a plant H^+^-ATPase can substitute for Pma1 in activating TORC1. This model, though largely hypothetical, is attractive, given the ideal position of fungal and plant H^+^-ATPases to sense both cytosolic ATP levels and the H^+^ influx coupled to active nutrient transport. It has been shown, furthermore, that the activity of plant H^+^-ATPases is regulated by practically all factors known to control plant growth, including those more recently shown to control TORC1 in plant cells^14^. A well-illustrated example of such a factor is auxin, which stimulates the activity of plant H^+^-ATPases by increasing phosphorylation of their penultimate Thr^43,44^, and which also promotes cell expansion and TORC1 activation^37^. Lastly this model, if valid, could also shed new light on the historical acid growth theory, by proposing that activation of H^+^-ATPases under acidic conditions increases cell growth both through H^+^ export (causing cell-wall loosening) and via TORC1 activation.

In conclusion, our results show that the *N. plumbaginifolia* PMA2 H^+^-ATPase can substitute for Pma1 in stimulating yeast TORC1 in response to an H^+^ influx or increase, and that this requires association of its cytosolic C-tail with 14–3–3 proteins. It will therefore be interesting to determine whether this property of PMA2 observed in yeast reflects an equivalent role naturally ensured by this H^+^-ATPase in plant cells.

## Methods

### Yeast strains, plasmids, and growth conditions

Strain JX023 (*GAL1p-PMA1 pma2Δ ura3 leu2*) used in this study derives from the Σ1278b wild type^7^. Strain CF108 is the equivalent strain in which the *BMH1* gene has been deleted. The plasmids used in this study are listed in Table 1. The *ura3* and *leu2* mutations present in the strains used were complemented by transformation with plasmids. The oligonucleotides used to isolate novel plasmids and strains are available on request. Cells were grown at 29 °C on a minimal medium buffered at pH 6.1^45^, with glucose (Gluc) (3% w/v) or galactose (Gal) (3% w/v) as a carbon source. The nitrogen sources added to the growth media were NH_4_^+^, as (NH_4_)_2_SO_4_ (20 mM), or proline (10 mM). When indicated, rapamycin (Rap) was added at 200 ng/ml. In all experiments, cells were examined or collected during exponential growth, a substantial and regular number of generations after seeding. In our experience, these precautions and the use of a buffered minimal medium considerably improve the reproducibility of results between biological replicates^46^. Comparative analyses of growth were performed by cultivating cells in a Greiner 24-well microplate incubator coupled to a SYNERGY™ multi-mode reader (BioTek Instruments).

**Table 1:** Plasmids used in this study.

Plasmid	Description	Reference or source
pFL38	*CEN-ARS (URA3)*	^51^
pFL36	*CEN-ARS (LEU2)*	^51^
pAS103	*YEp-HA-NPR1 (URA3)*	^29^
pHl-U	*YEp-ADH1p-pHluorin (URA3)*	^52,53^
pPS15-P1	*YCp-(Sc)PMA1 (LEU2)*	^54^
pPMA4^882ochre^	*YEp-PMA1p-(Np)PMA4*^*882ochre*^* (LEU2)*	^11^
pPMA2^E14D^	*YEp-PMA1p-6xHis-(Np)PMA2*^*E14D*^* (LEU2)*	^11^
pES150	*YEp-PMA1p-6xHis-(Np)PMA2*^*E14D-Δ(887-956)*^* (LEU2)*	This study
pCF053	*YEp-PMA1p-6xHis-(Np)PMA2*^*E14D-Δ(880-956)*^* (LEU2)*	This study
pCF052	*YEp-PMA1p-6xHis-(Np)PMA2*^*E14D-Δ(865-956)*^* (LEU2)*	This study
pES162	*YEp-PMA1p-6xHis-(Np)PMA2*^*E14D-T955A*^* (LEU2)*	This study
pES164	*YEp-PMA1p-6xHis-(Np)PMA2*^*E14D-S938A*^* (LEU2)*	This study
pES166	*YEp-PMA1p-6xHis-(Np)PMA2*^*E14D-S938D*^* (LEU2)*	This study
pES168	*YEp-PMA1p-6xHis-(Np)PMA2*^*E14D-T931A*^* (LEU2)*	This study
pES170	*YEp-PMA1p-6xHis-(Np)PMA2*^*E14D-T931D*^* (LEU2)*	This study
pES173	*YCp-6xHis-5xGA-(Sc)PMA1 (LEU2)*	This study

### Yeast cell extracts, immunoblotting, and pull-down experiments

For western blot analysis, crude cell extracts were prepared as previously described^47^. Proteins were transferred to a nitrocellulose membrane (Schleicher and Schuell; catalog number NBA085B) and probed with mouse anti-hemagglutinin (anti-HA) (12CA5; Roche), anti-yeast 3-phosphoglycerate kinase (anti-PGK) (Invitrogen), anti-polyhistidine (H1029; Sigma-Aldrich), rabbit anti-phospho-Thr^737^-Sch9^7^, anti-Sch9_Total_ (a gift from Robbie Loewith), or anti-14–3–3 (a gift of Marc Boutry). Primary antibodies were detected with horseradish-peroxidase-conjugated anti-mouse or anti-rabbit immunoglobulin G secondary antibodies (GE Healthcare), followed by enhanced chemiluminescence (Roche; catalog number 12 015 196 001). Each western blot was prepared at least twice. A representative experiment is presented. In pull-down experiments, exponentially growing cells were first resuspended in lysis buffer (50 mM Tris–HCl, pH 7.2, 600 mM NaCl, 1% Nonidet P-40) supplemented with complete EDTA-free protease inhibitor cocktail tablets (Roche), 1 mM phenylmethylsulfonyl fluoride (PMSF), 25 mM N-ethylmaleimide, 50 μM protease inhibitor MG-132 (Sigma-Aldrich), and 0.5% sodium deoxycholate. The cells were lysed by vortexing in the presence of glass beads, and cell extracts were incubated for 30 min on ice and centrifuged for 30 min at 3000 rpm. The polyhistidine-tagged Pma1 or PMA2^E14D^ proteins (and variants) were purified from the lysates with Dynabeads (Invitrogen; catalog number 10103D).

### Measurements of cytosolic pH

Intracellular pH was measured with pHluorin as previously described^7^. Data points represent averages of three biological replicates; error bars represent standard deviations (SD).

### Measurements of radiolabeled β-alanine uptake

Accumulation of [^14^C]-labeled β-alanine (Hartmann analytic) was measured at the indicated time points as previously described^48,49^. Data points represent averages of three biological replicates; error bars represent standard deviations (SD).

### Measurements of acidification of external medium

A previously published protocol^50^ with minor modifications was applied. Yeast cells were grown on Gluc NH_4_^+^ medium to OD660 ~ 0.2 and 100 ml was harvested, washed three times with 10 ml cold water, resuspended in a vial containing 25 ml of 0.2 mM MES buffer at pH 6.1 (KOH), and preheated at 29 °C. With a pH electrode and a shaking incubator at 29 °C, the pH was recorded for 20 min (time needed to stabilize the external pH) prior to addition of Gluc (200 mM) and KCl (20 mM) at time 0. The pH was then recorded for another 15 min. Data points represent averages of three biological replicates and error bars represent standard deviations (SD).

## Supplementary Information

Supplementary Information

## Data Availability

All data are contained within the manuscript.

## Abbreviations

BAF: Bafilomycin A
C-tail: Carboxy-terminal tail
FCCP: Carbonyl cyanide-4-(trifluoromethoxy)phenylhydrazone
Gap1: General amino-acid permease 1
Gluc: Glucose medium
RAP: Rapamycin
TORC1: Target of rapamycin complex 1
β-Ala: β-Alanine
