# Supplementary material for: A plant plasma-membrane H+-ATPase promotes yeast TORC1 activation via its carboxy-terminal tail
Source: Sci Rep. 2021 Feb 26;11:4788. doi: 10.1038/s41598-021-83525-1 (PMC7910539; doi:10.1038/s41598-021-83525-1)
Supplement: Supplementary file 1 — Supplementary Information [file 41598_2021_83525_MOESM1_ESM.pdf]

A plant plasma-membrane H^+-ATPase promotes yeast TORC1 activation via its carboxy-terminal tail

Elie Saliba, Cecilia Primo, Nadia Guarini and Bruno André

Supplementary information

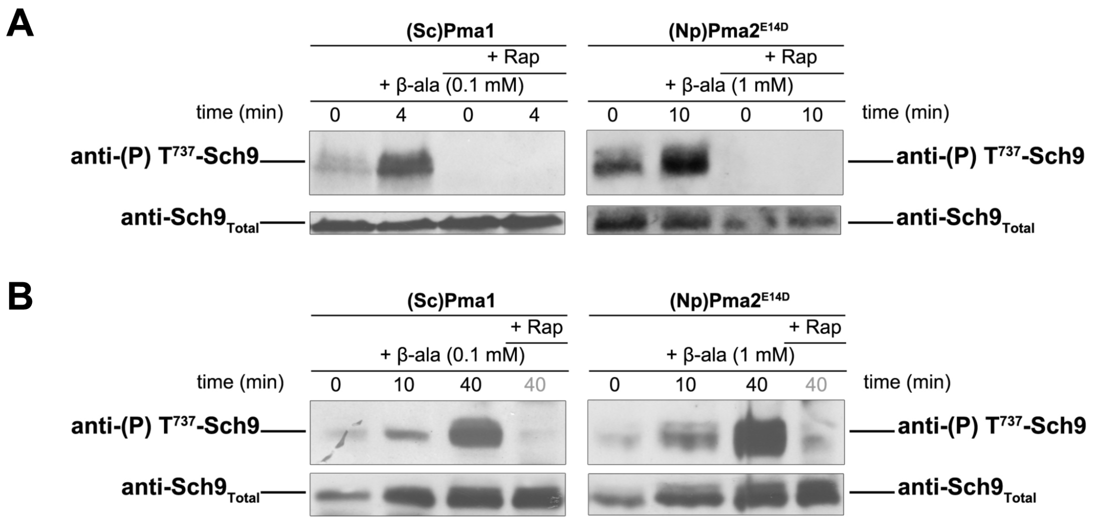

Figure S1. TORC1 activation upon H^+-coupled β-alanine uptake in cells expressing the endogenous Pma1 or the plant PMA2^{E14D} H^+-ATPase is inhibited by rapamycin

GAL1p-PMA1 pma2Δ cells expressing, from two plasmids, either (Sc)Pma1 or (Np)PMA2^{E14D} along with HA-NPR1, were grown on Gluc NH_4^+ medium. After a shift to Gluc proline medium for four hours, β-alanine (0.1 or 1 mM) was added to the medium. Cells were collected before and several time intervals after addition of β-alanine. Crude extracts were prepared and immunoblotted with anti-(P) T^{737}-Sch9 and anti-Sch9$_{Total}$ antibodies. (A) Part of each culture was treated for 30 min with rapamycin (Rap) before adding β-alanine. (B) After addition of β-alanine for 10 min, part of the culture was treated with rapamycin (Rap) for 30 minutes.

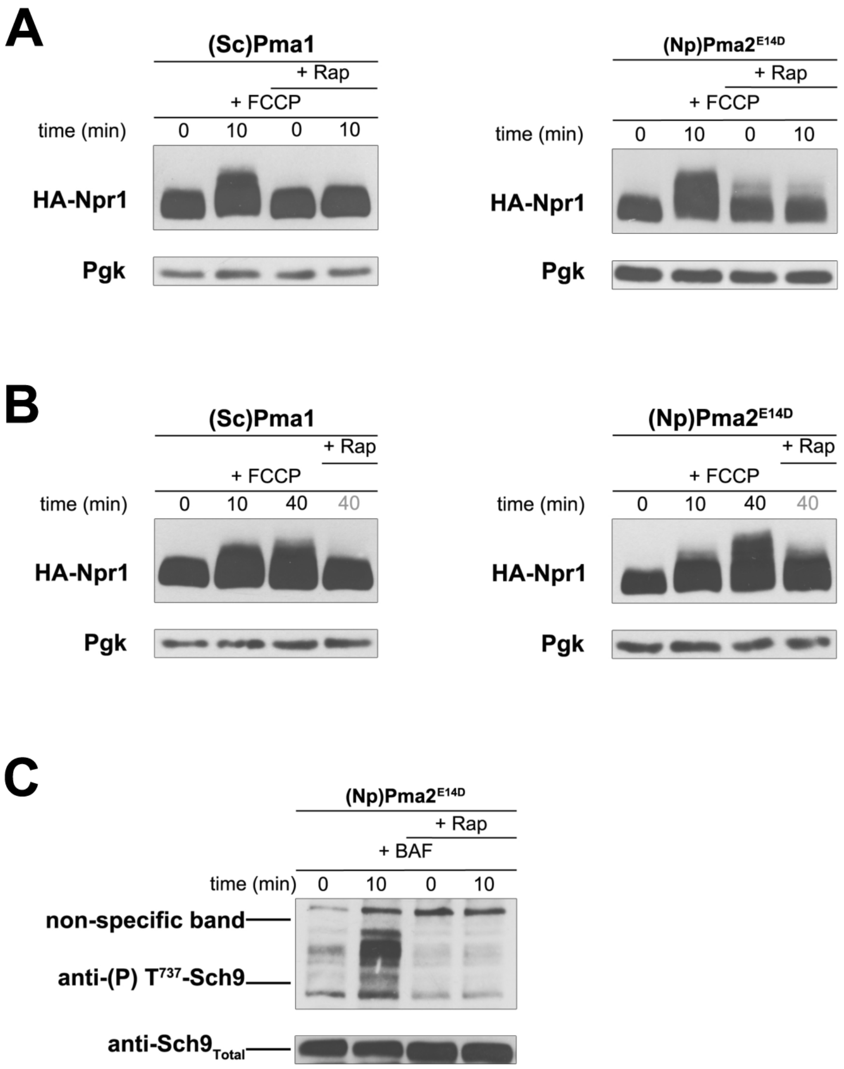

Figure S2. TORC1 activation upon FCCP or bafilomycin A addition to cells expressing the endogenous Pma1 or the plant PMA2^{E14D} H^+-ATPase is inhibited by rapamycin

GAL1p-PMA1 pma2Δ cells expressing, from two plasmids, either (Sc)Pma1 or (Np)PMA2^{E14D} along with HA-NPR1, were grown on Gluc NH_4^+ medium. After a shift to Gluc proline medium for four hours, cells were collected before and several time intervals after addition of FCCP (20 µM) (A, B) or bafilomycin A (BAF) (1 µM) (C). Part of each culture was treated for 30 minutes with rapamycin (Rap) before (A, C) or after (B) the treatment. As cells treated with FCCP typically responded less to rapamycin added afterwards, a higher concentration of rapamycin (3 µgr/ml) was used (B).

Figure S3. Original blots

Figure 1D

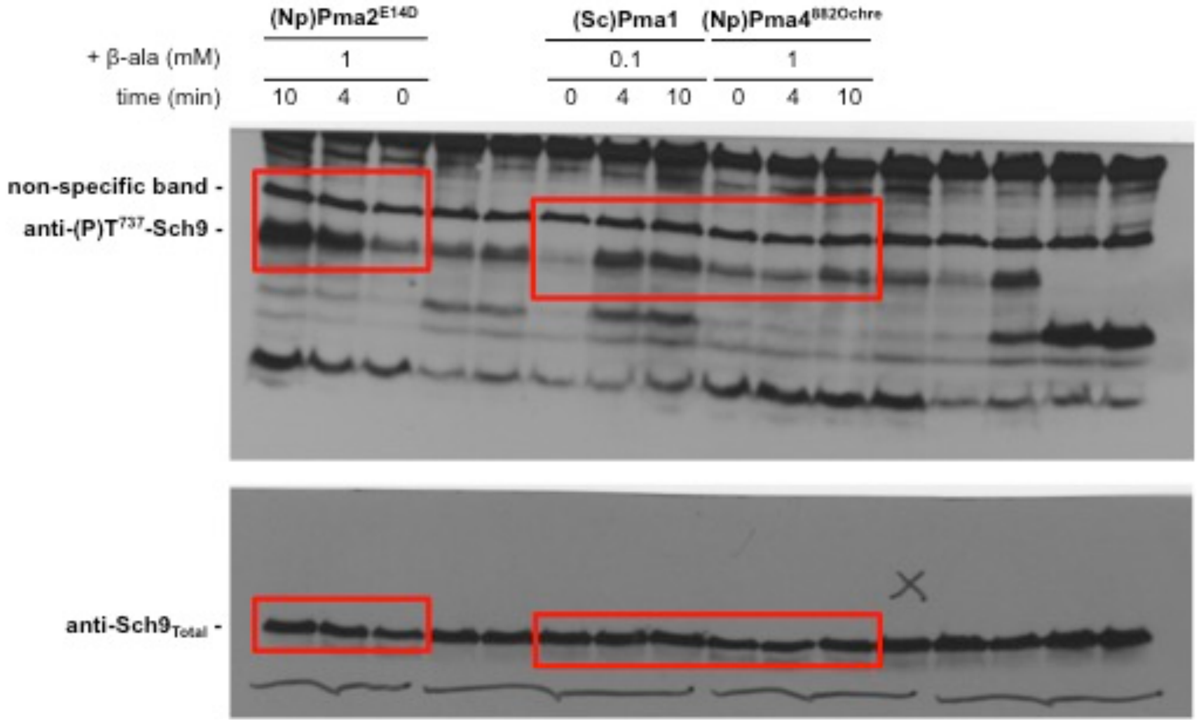

Figure 2B

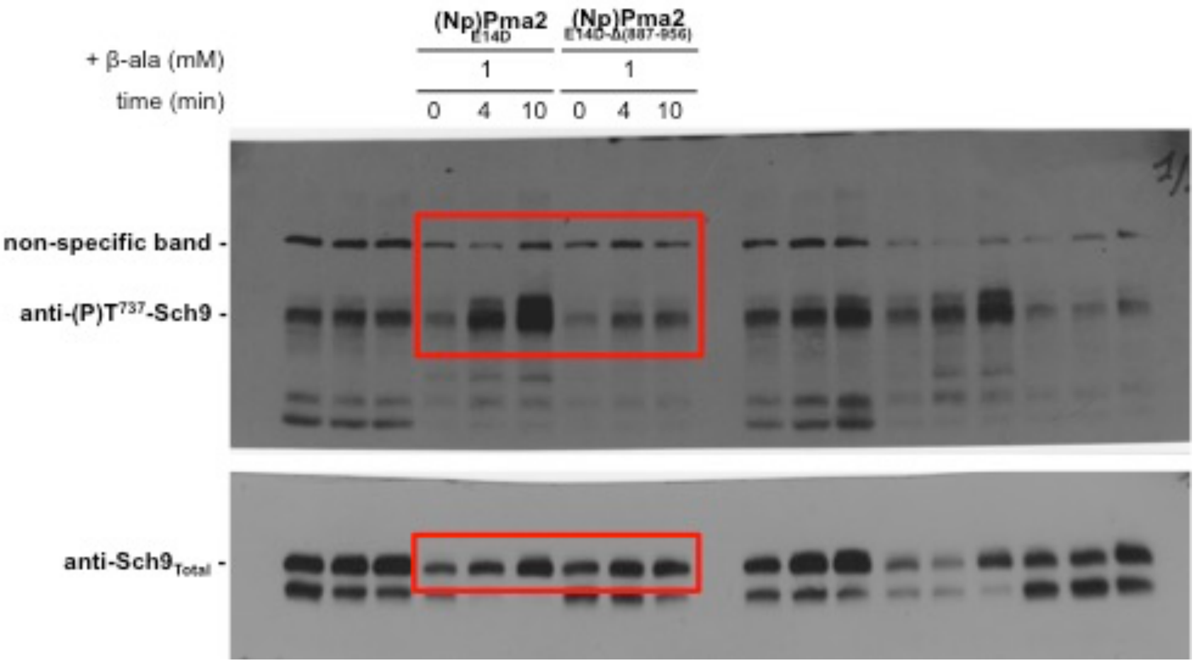

Figure 2D

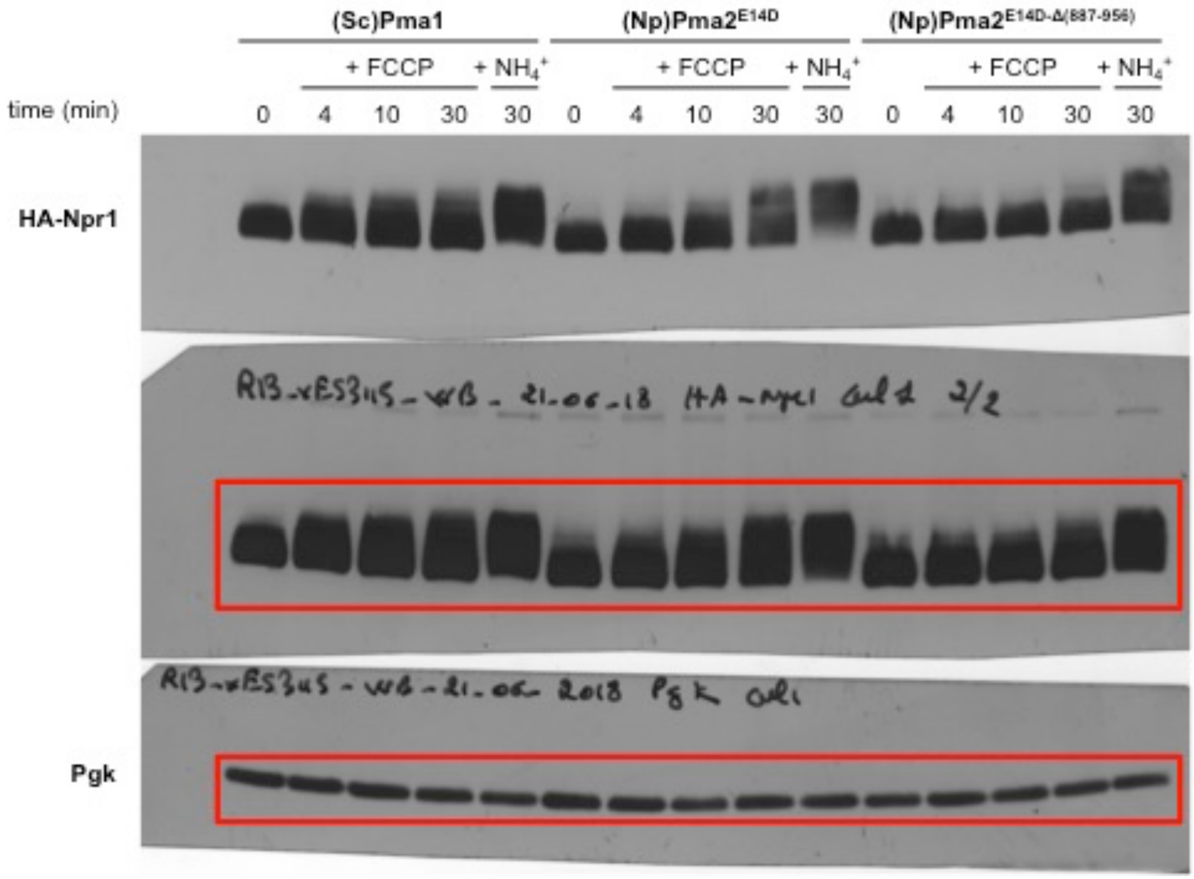

Figure 2F

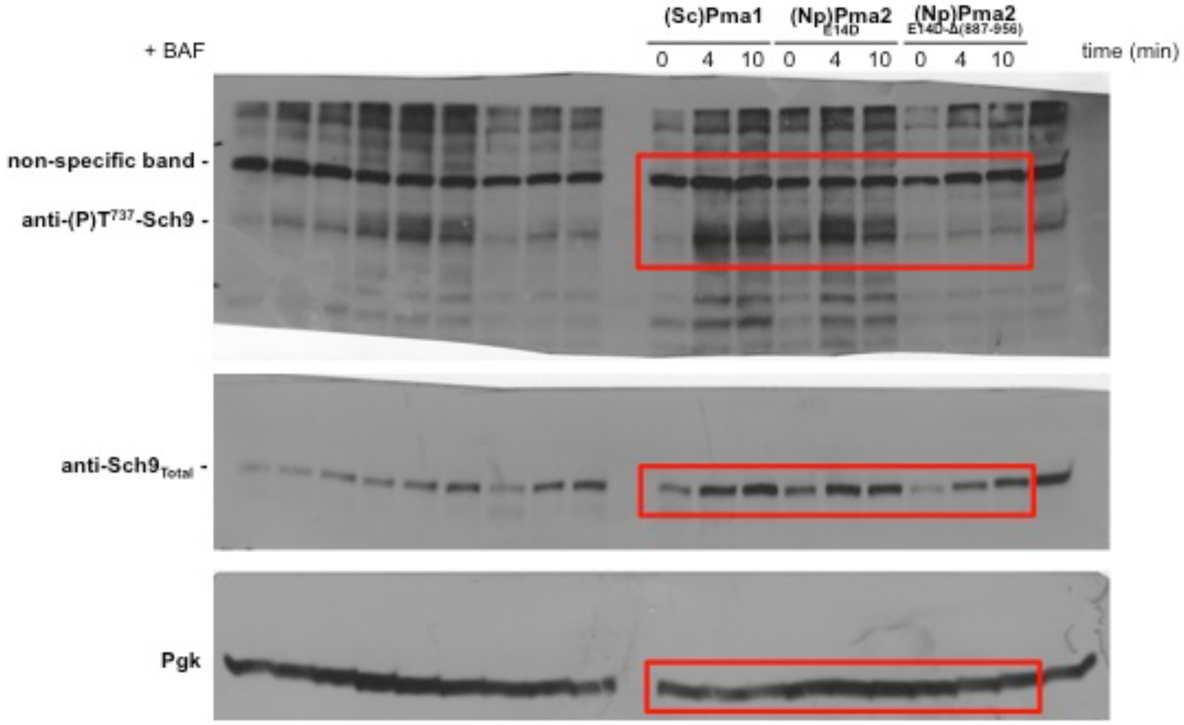

Figure 2G

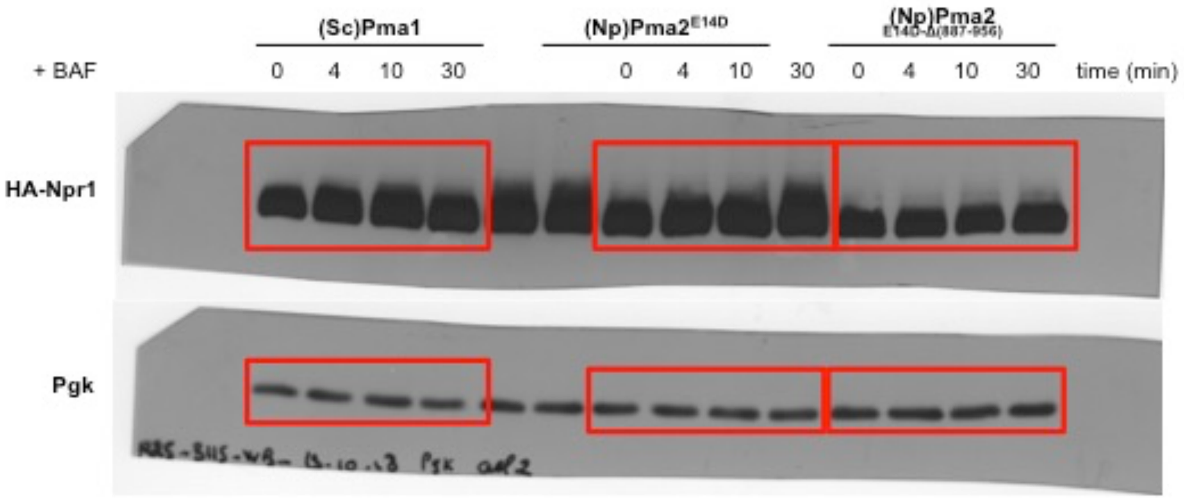

Figure 3B

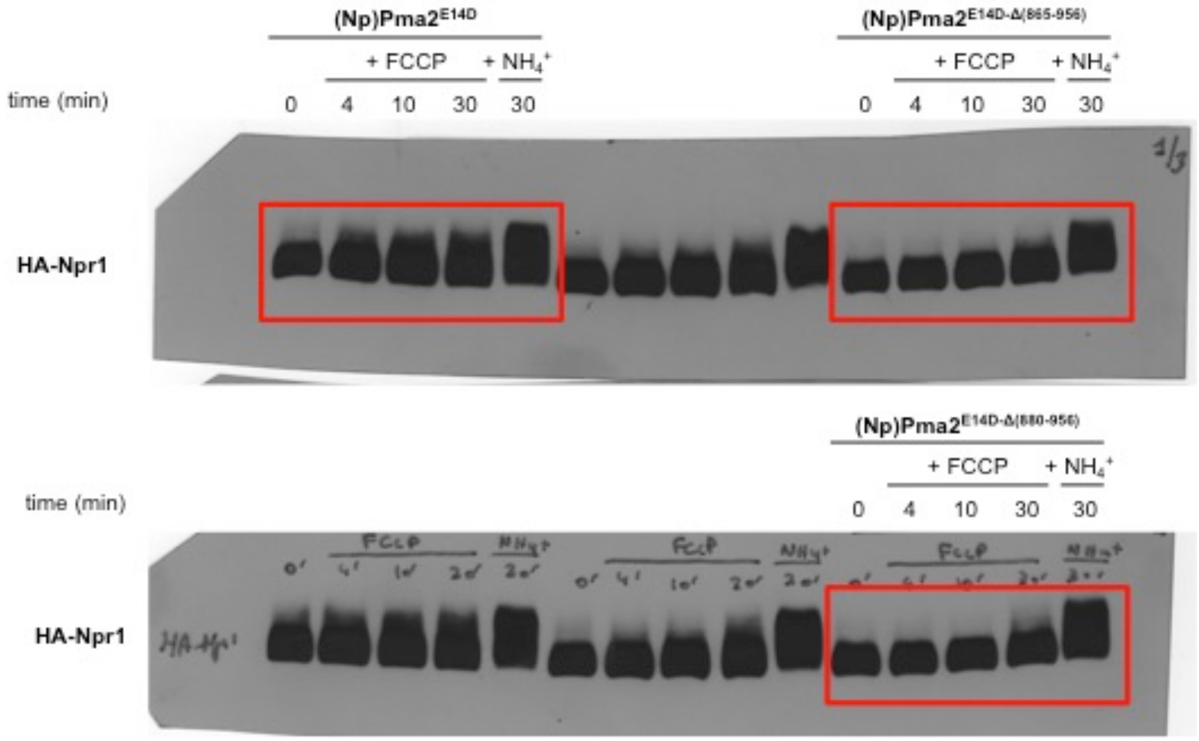

Figure 3E

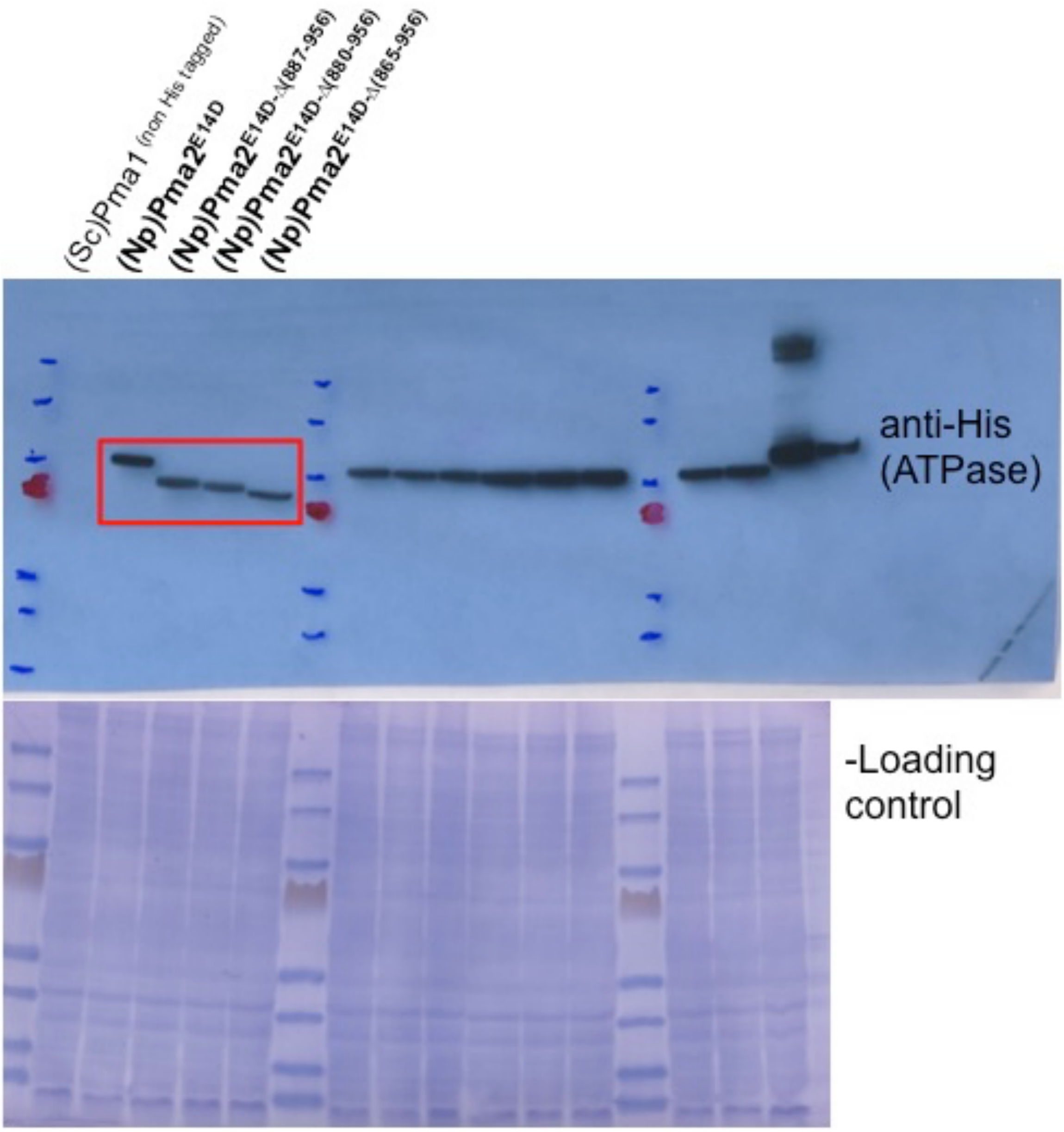
(Sc)Pma1 (non His tagged)
(Np)Pma2E14D
(Np)Pma2E14D-Δ(887-956)
(Np)Pma2E14D-Δ(880-956)
(Np)Pma2E14D-Δ(865-956)
anti-His
(ATPase)
-Loading
control

Figure 4D

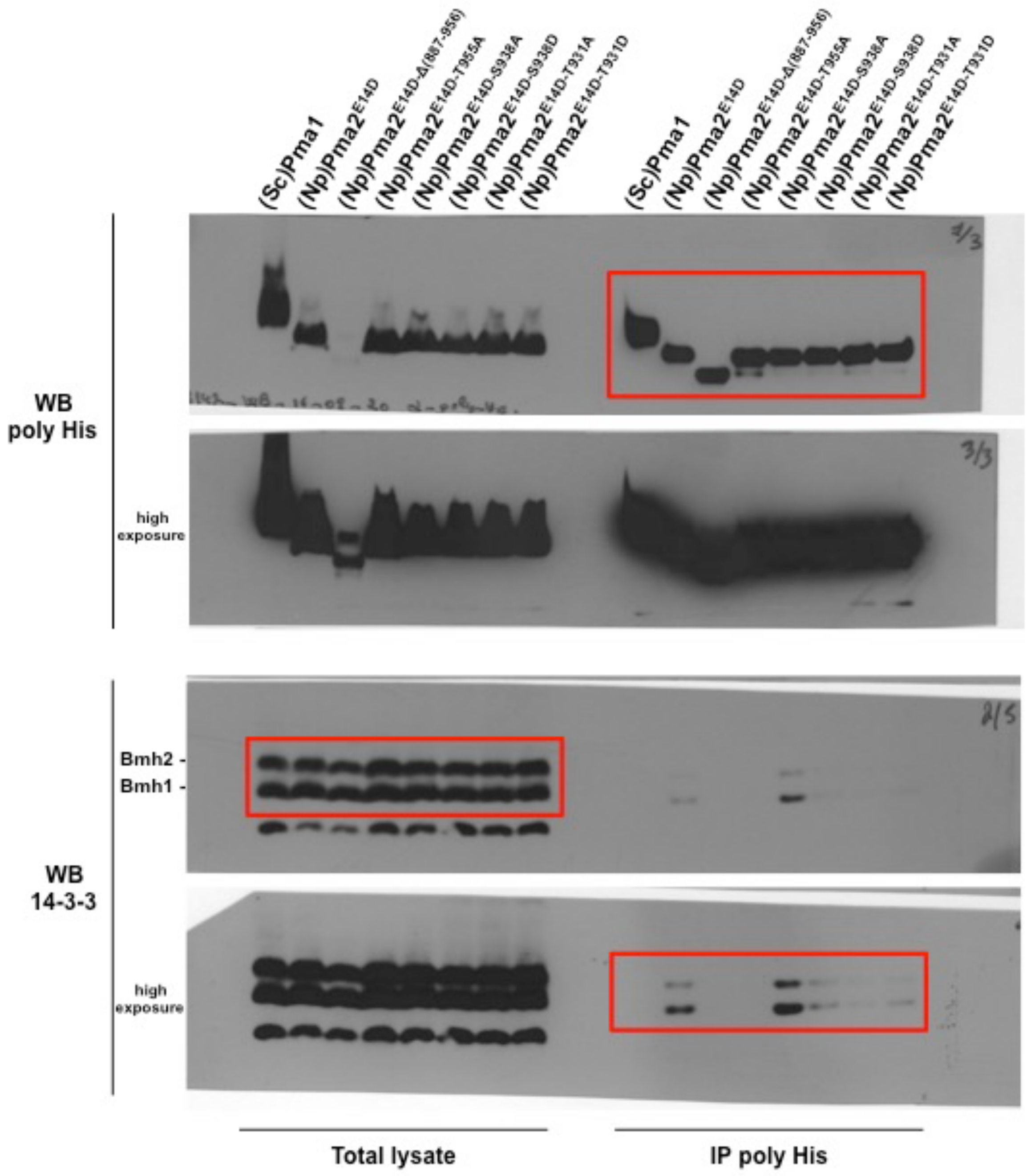

(Sc)Pma1
(Np)Pma2E14D
(Np)Pma2E14D-Δ(887-956)
(Np)Pma2E14D-T955A
(Np)Pma2E14D-S938A
(Np)Pma2E14D-S938D
(Np)Pma2E14D-T931A
(Np)Pma2E14D-T931D
WB
poly His
high
exposure
WB
14-3-3
Bmh2 -
Bmh1 -
high
exposure
Total lysate
IP poly His

Figure 4E

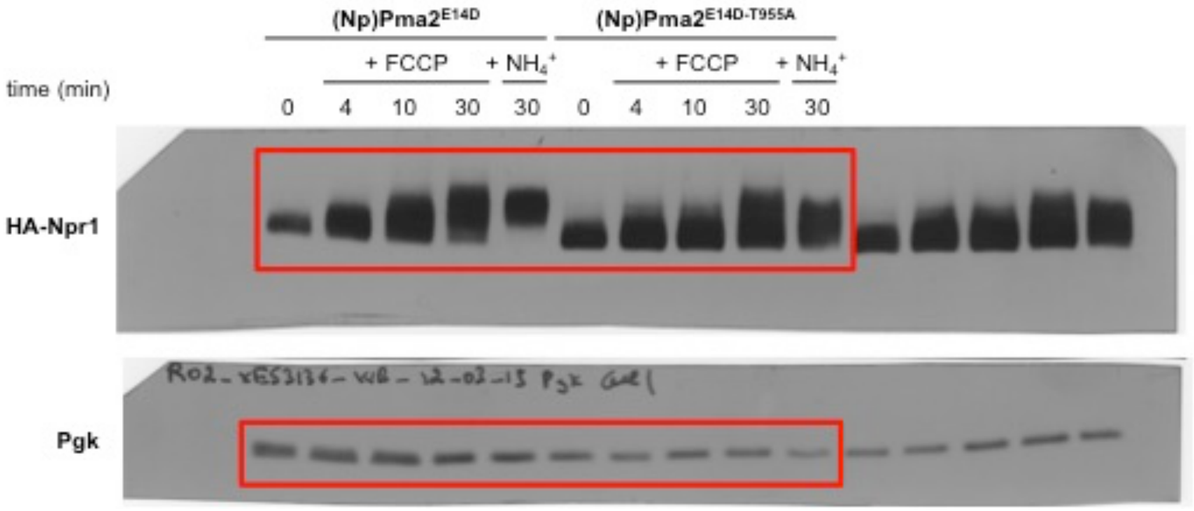

Figure 4F

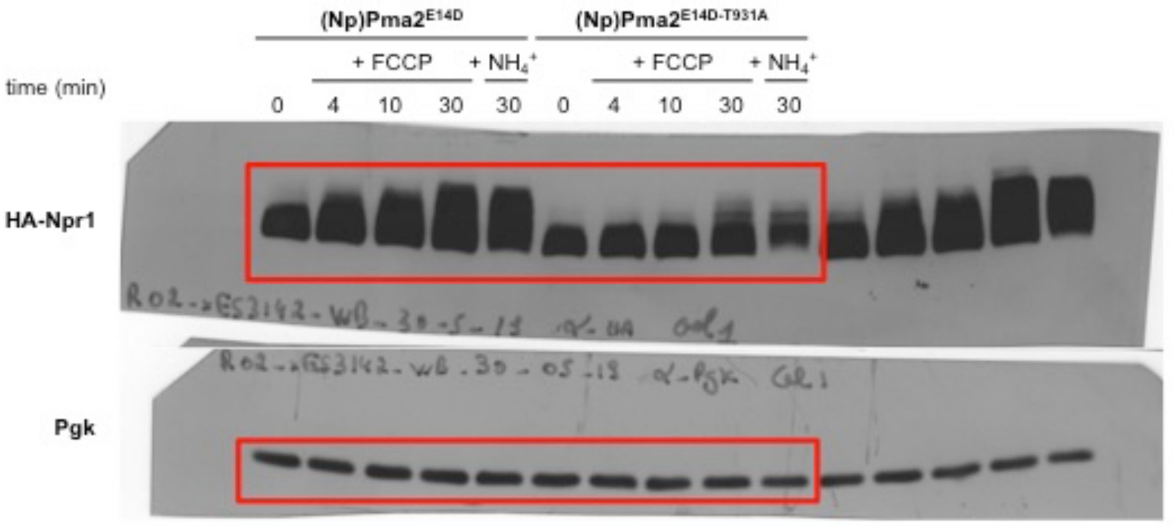

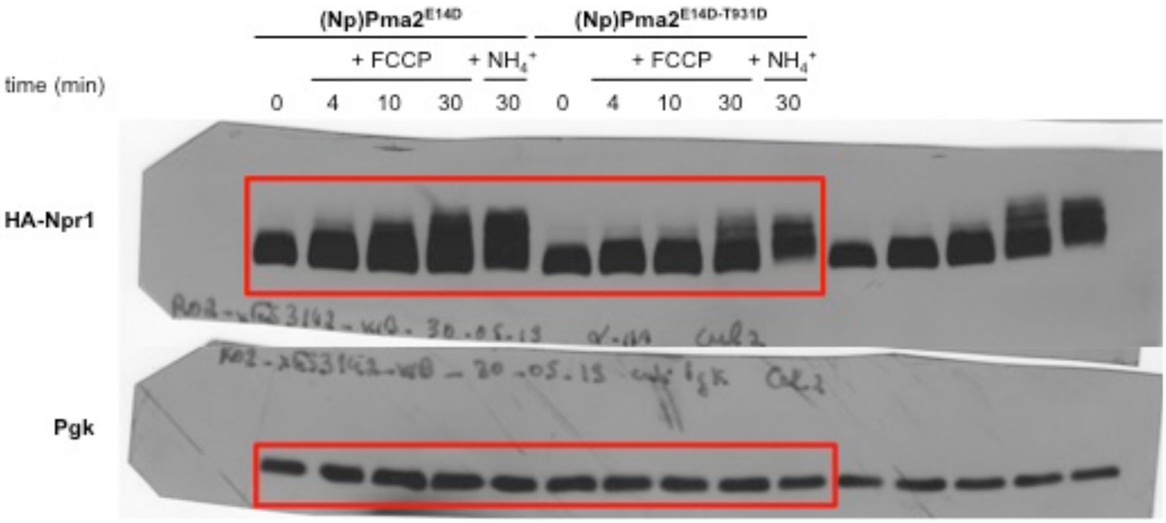

Figure 4G

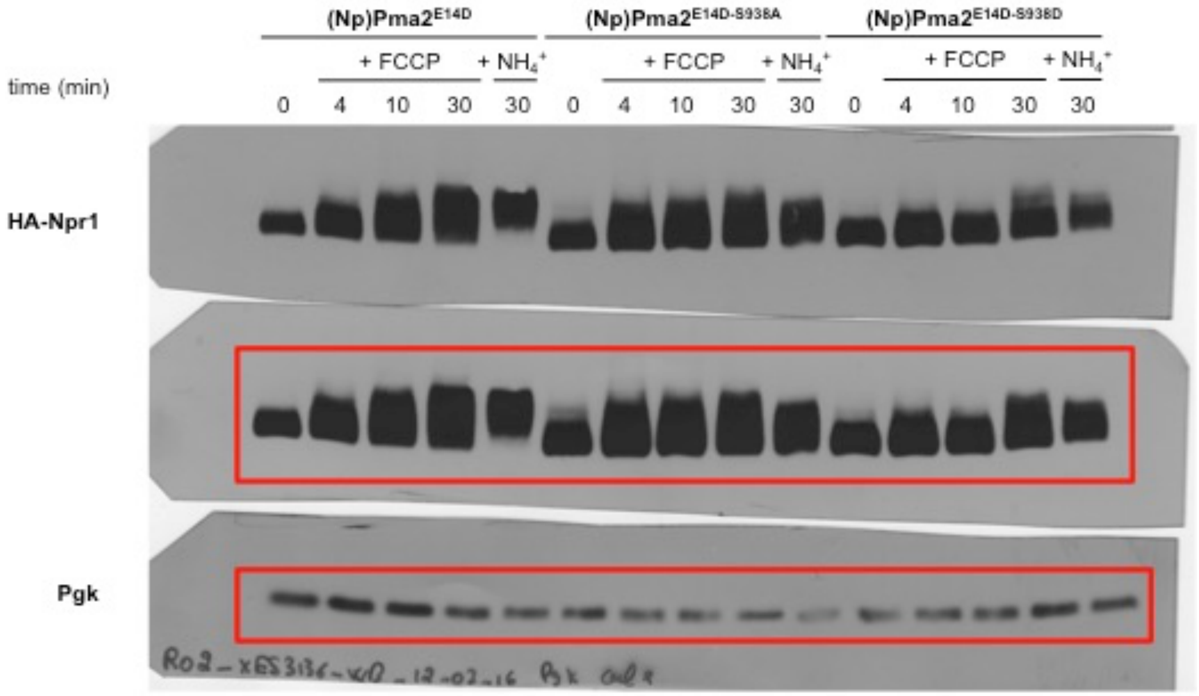
(Np)Pma2E14D
(Np)Pma2E14D-S938A
(Np)Pma2E14D-S938D
+ FCCP
+ NH4+
time (min)
0 4 10 30 30 0 4 10 30 30 0 4 10 30 30
HA-Npr1
Pgk

Figure 5C

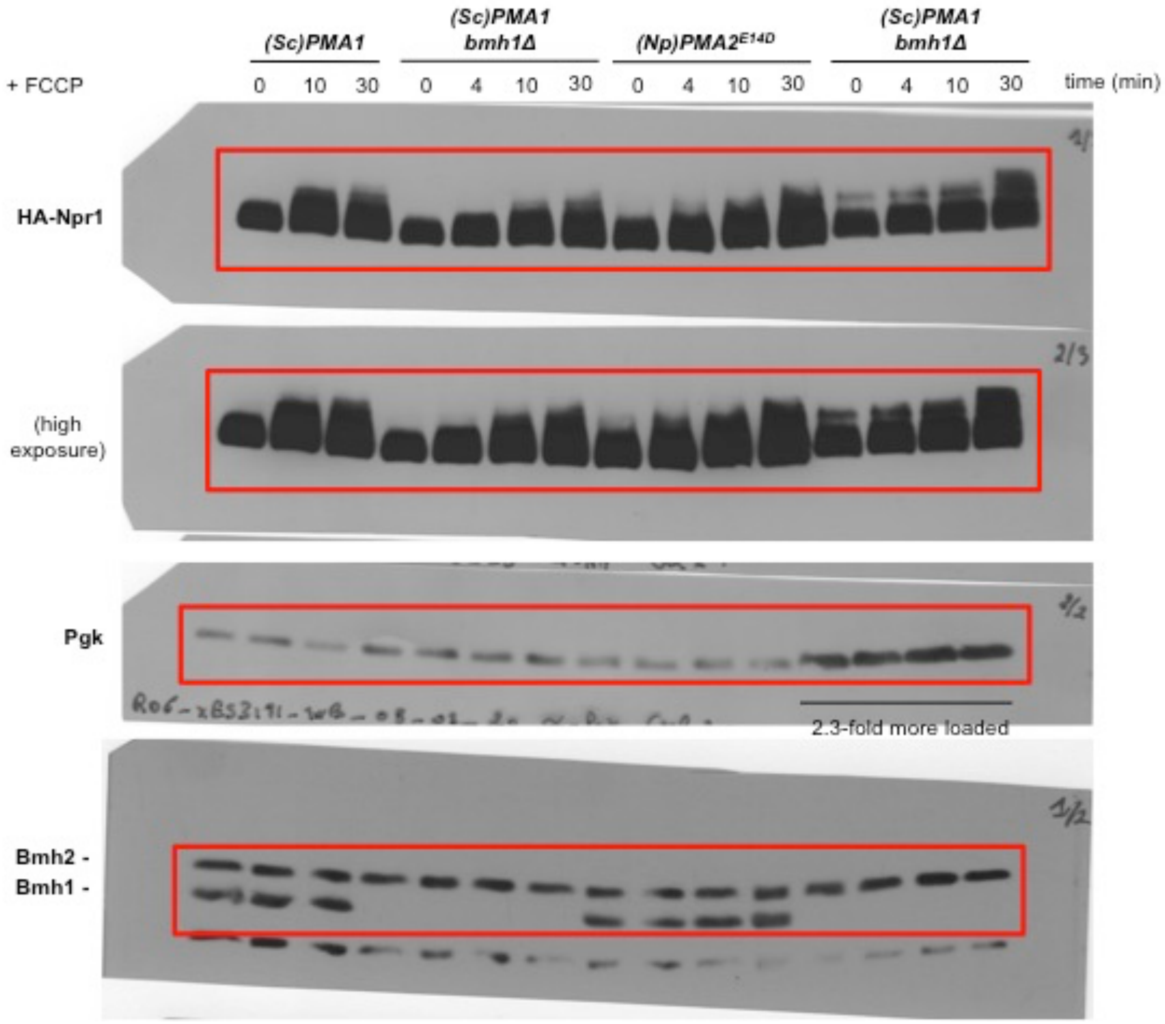
(Sc)PMA1
(Sc)PMA1 bmh1Δ
(Np)PMA2E14D
(Sc)PMA1 bmh1Δ
+ FCCP
0 10 30 0 4 10 30 0 4 10 30 0 4 10 30
time (min)
HA-Npr1
(high exposure)
Pgk
2.3-fold more loaded
Bmh2 -
Bmh1 -

Figure S1A, left part

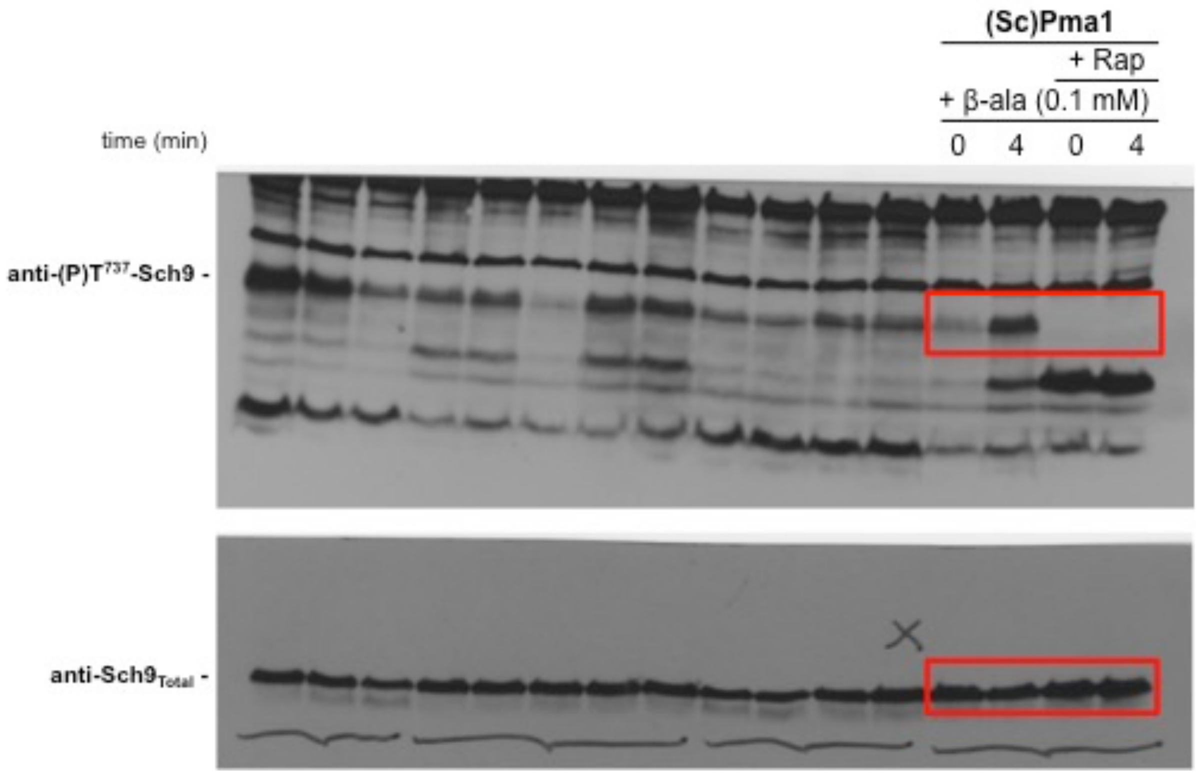

Figure S1A, right part

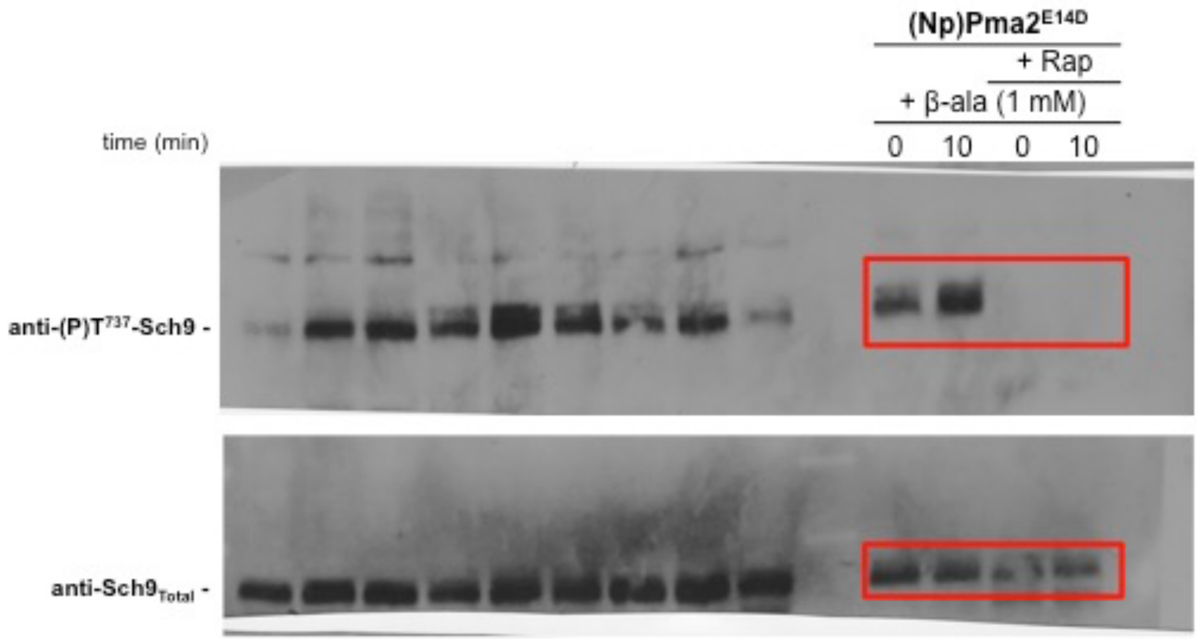

Figure S1B, left part

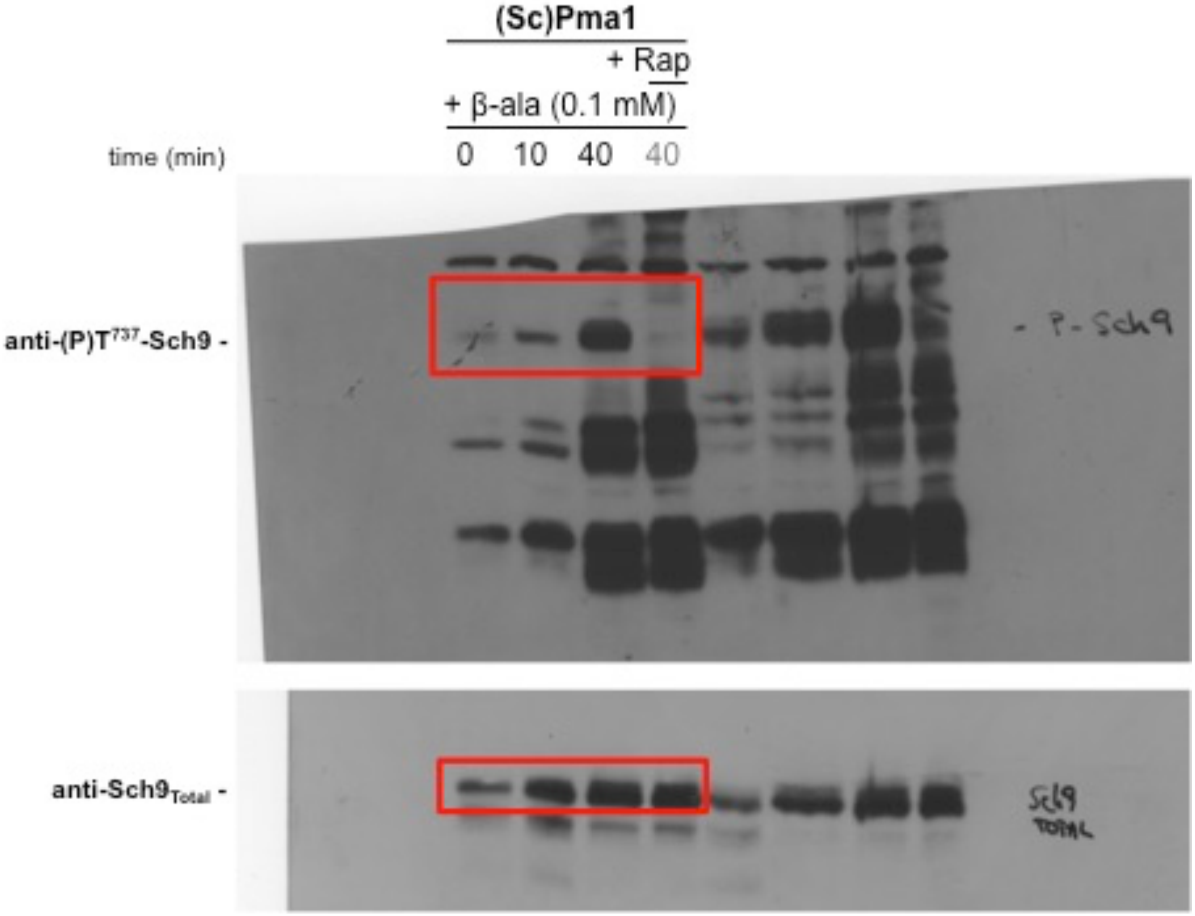

Figure S1B, right part

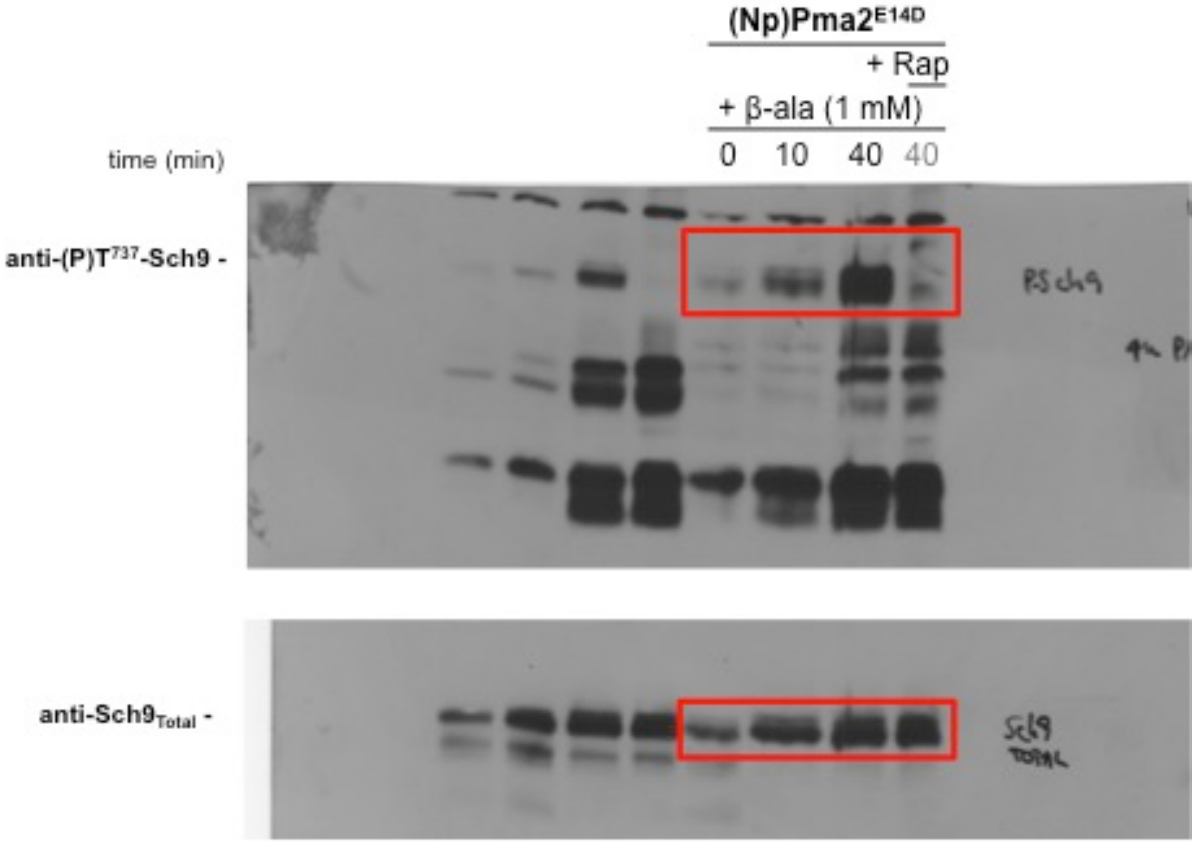

Figure S2A

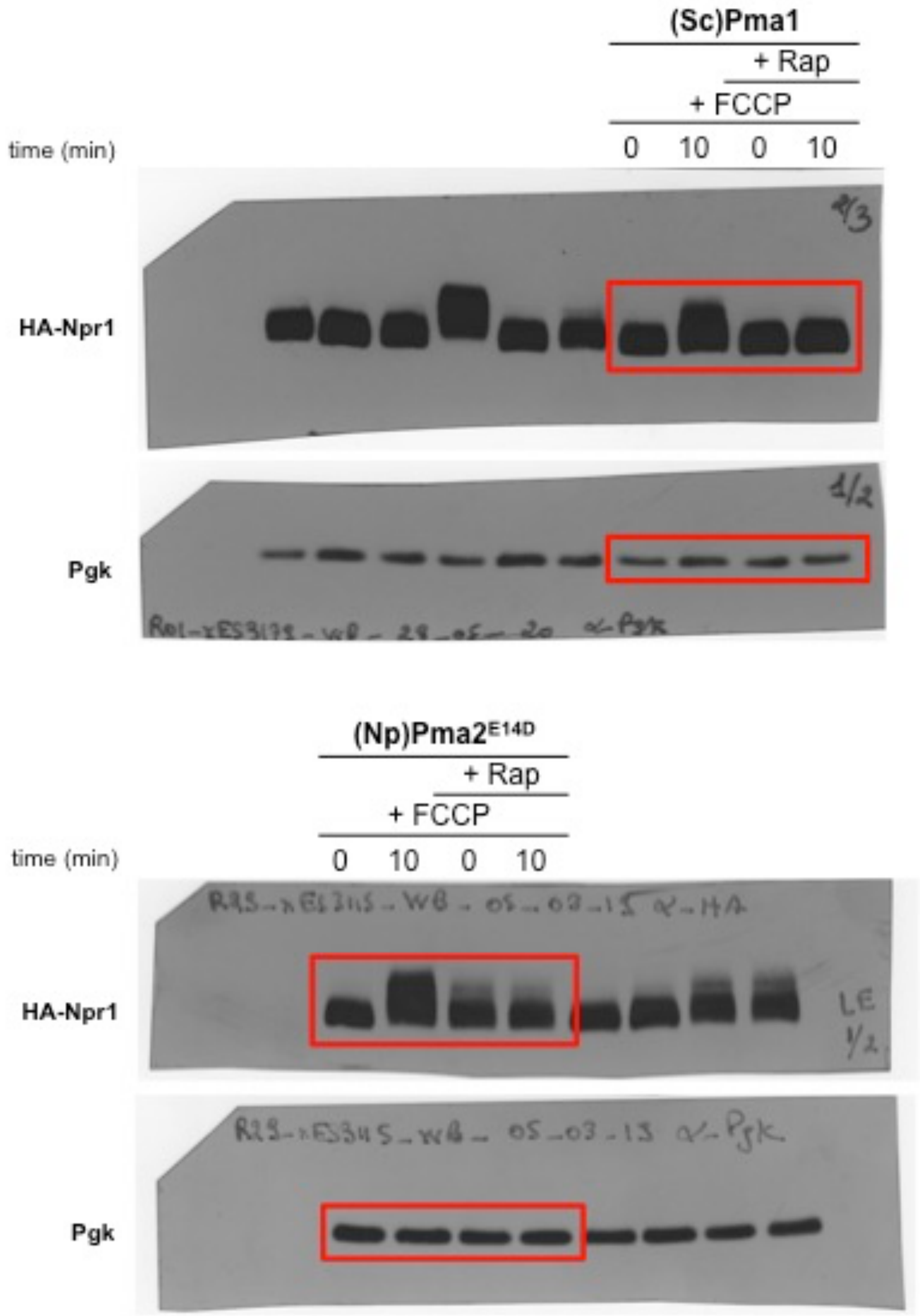

(Sc)Pma1
+ Rap
+ FCCP
time (min)
0
10
0
10
HA-Npr1
Pgk
(Np)Pma2E14D
+ Rap
+ FCCP
time (min)
0
10
0
10
HA-Npr1
Pgk

Figure S2B

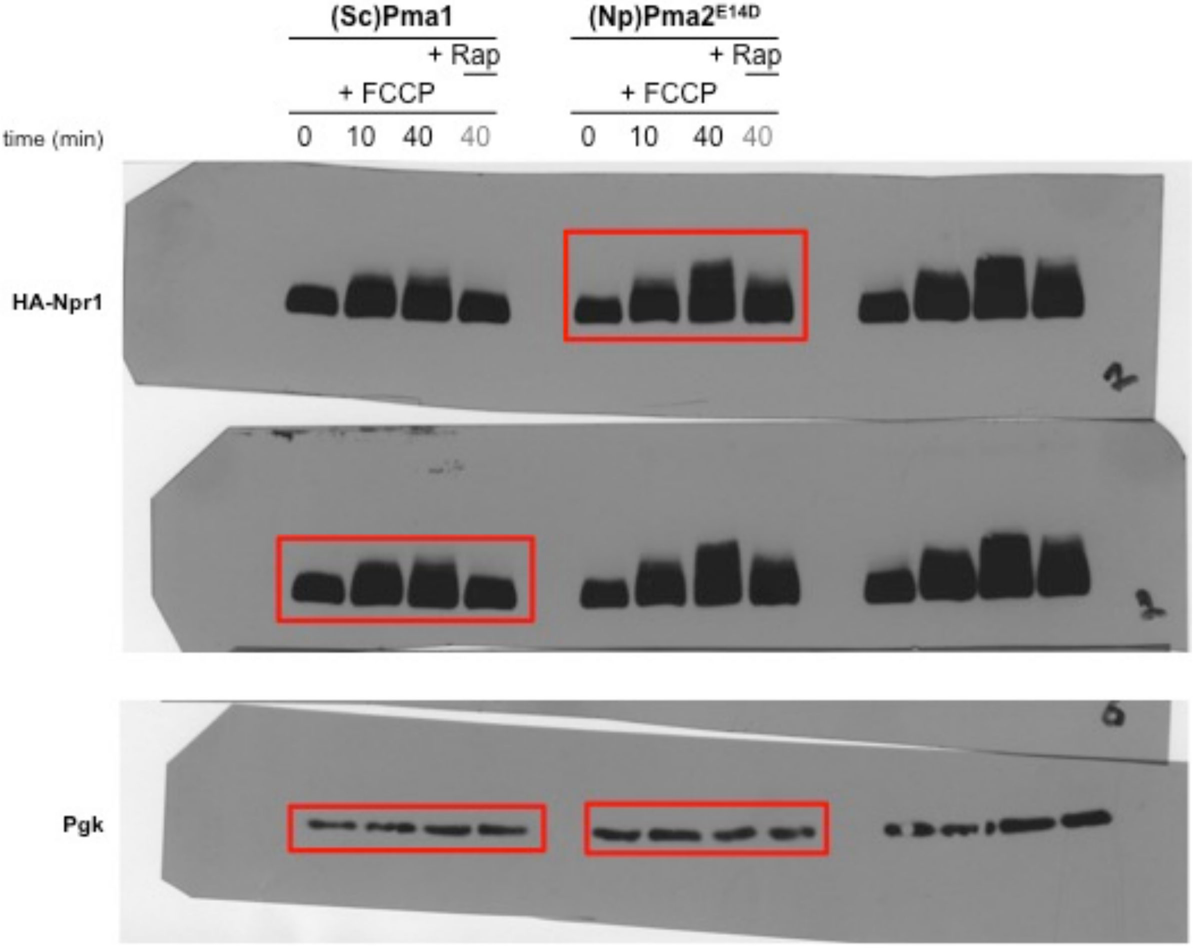

(Sc)Pma1
(Np)Pma2E14D
+ Rap
+ Rap
+ FCCP
+ FCCP
time (min)
0 10 40 40
0 10 40 40
HA-Npr1
Pgk

Figure S2C

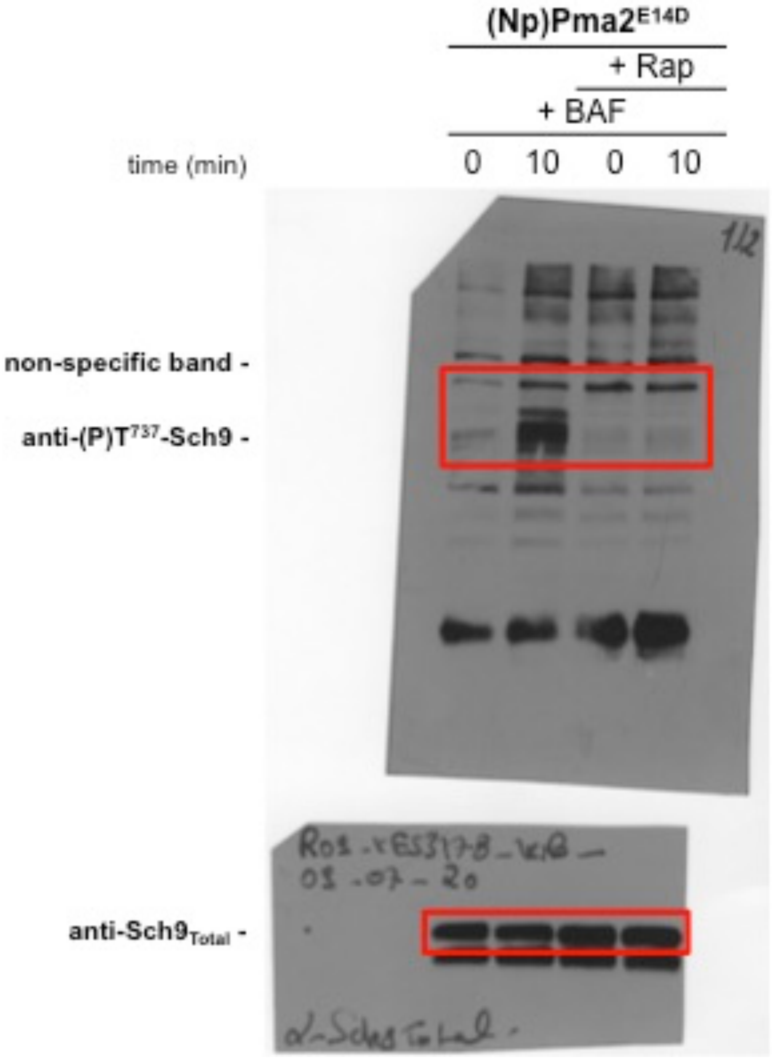
(Np)Pma2^E14D
+ Rap
+ BAF
time (min)
0 10 0 10
non-specific band -
anti-(P)T^737-Sch9 -
anti-Sch9_Total -
